# Heart Meets Brain: Insights into Neurocardiac Pathophysiology

**DOI:** 10.3390/pathophysiology33030058

**Published:** 2026-08-10

**Authors:** Berk Rasheed, Jacob Freed, Ruhi Parikh, Aman Singh, Krishna K. Singh

**Affiliations:** 1 College of Medicine, University of Illinois Chicago, Rockford, IL 61107, USA; jfree12@uic.edu; 2 Department of Medical Biophysics, Western University, London, ON N6A 5C1, Canada; rparik5@uwo.ca (R.P.); asin24@uwo.ca (A.S.)

**Keywords:** neurocardiology, bidirectional heart–brain axis, sympathetic hyperactivity, cerebral blood flow, neuroinflammation

## Abstract

The intricate relationship between the heart and the brain has transitioned from historical, cardio-centric theories to a modern, circuit-based, bi-directional framework known as neurocardiology. This review provides a comprehensive anatomical, physiological, and clinical synthesis of the bidirectional heart–brain axis, highlighting how disruptions in one organ systematically impact the other. Structurally, the heart–brain axis operates as a closed-loop feedback network. Vagal parasympathetic control dominates at rest, driven by an asymmetrical fiber distribution where sensory afferents actively supply interoceptive data to the brainstem. Hemodynamically, cerebral blood flow delivery is linked to cardiac output in an age-dependent fashion with the related vascular dysfunction. Clinically, brain-to-heart pathologies like acute brain injury trigger life-threatening catecholamine surges, neuroinflammation, and myocardial stunning, including Takotsubo cardiomyopathy. Conversely, heart-to-brain pathways reveal that atrial fibrillation and heart failure can independently trigger cardioembolic stroke, long-term neuroinflammation, and a profound, progressive burden of cognitive decline. The heart and brain are inexorably linked through complex structural, mechanical, and paracrine pathways. Ameliorating clinical outcomes demands integrated cross-specialty diagnostic screening, digital rhythm monitoring, and holistic neurocardioprotective therapeutic strategies.

## 1. Introduction

### 1.1. Historical Perspective: Concept of the Heart–Brain Connection

In the past two millennia, various links between the heart and brain have been disputed and explored, primarily on the premise of emotions and intelligence belonging to one system or the other. Over this period, an integrative empirical approach became rooted in the framing of longstanding theories assessing the anatomical and functional bases of the brain and heart, but the viewpoint in antiquity until the early 20th century was almost exclusively cardio-centric [1]. The historic transition from cardio-centric accounts of the mind and emotion towards a more mechanistic model that comprised circuit-based central neural controls with the measurement of cardiovascular dynamics occurred in the early 18th century. Some of the earliest accounts of this investigatory framework between the nervous system and cardiovascular system emerged through the work of Stephen Hales in 1733, where he described that the beat-to-beat interval and arterial pressure varied across the respiratory cycle, promoting the theorization that cardiac regulation was not wholly independent [1,2,3]. It was not until 1845 that a study by the Weber brothers introduced a foundational neurophysiological concept for cardiac control, where they demonstrated that the heart could be arrested via vagal stimulation [4]. Furthermore, Ludwig in 1847 built on the foundation introduced by Hales by documenting pulse acceleration with inspiration and subsequent slowing with expiration, an early account of what is now recognized as respiratory sinus arrhythmia [5].

The early experimental work evolved the heart–brain connection to be brain-centered, and the early-to-mid 20th century work shaped this connection to be empirically supported through chemical neurotransmission, the mapping of afferent reflex arcs, and central autonomic regulation via hypothalamic and cortical systems [4,6,7]. Some of the earliest evidence of chemical neurotransmission was established by Loewi et al. in 1921 where they demonstrated that vagus stimulation slowed the heart rate and that transferring perfusate from these frog hearts to sinus hearts reproduced the bradycardia [8]. The active “substance”, initially described as vagustoff, was later identified as acetylcholine in the 1930s [6]. Adding to the sympathetic narrative, von Euler et al. in 1946 identified noradrenaline as the main constituent of signaling in the sympathetic nervous system (SNS) and reinforced the differentiation of adrenergic versus cholinergic neurons in neurocardiac signaling [9]. Various cortico-visceral theories also emerged in this era that placed cardiac visceral regulation under cortical control via afferent signaling and conditioned reflexes, extending the heart–brain connection into proposals that conceptualized disease pathogenesis. A prominent Soviet physiologist, Konstantin Bykov, theorized that visceral autonomic and endocrine functions were dictated via cortical structures through afferent signaling to higher nervous activity but with interoceptive communication [10]. Corticovisceral pathogeneses were proposed for various diseases such as hypertension and myocardial infarction, reflecting attempts to connect central regulation to cardiovascular pathology [10]. Afferent reflex physiology matured as a concrete, bidirectional pathway in which vascular-state signals (carotid sinus and carotid body) were localized as initiators of the reflex heart-inhibitory and vasodilatory responses [7,11].

These shifts in thinking helped build our understanding of the modern heart–brain connection, now referred to as “neurocardiology”, coined by Natelson [12]. Neurocardiology allowed for the recognition of intrinsic cardiac neural processing, which reframed the heart as containing local neural integration rather than being solely an effector of brainstem output [6,13,14]. The evidence for this framing was guided by Armour’s pioneering work in neurocardiology wherein he described the arrangement and architecture of afferent, efferent, and interneurons in epicardial ganglionic plexi as the heart’s “little brain” [13]. Thus, in the modern view, cardiac regulation is not only descending control from the brain to heart but also includes intrinsic cardiac neural processing (“little brain” concepts) and ascending sensory (afferent) signaling that shapes brain function through interoceptive pathways [6,13,15]. The heart–brain connection is now viewed as distributed, bidirectional, and quantifiable, explicitly integrating central autonomic networks, peripheral autonomic pathways, intrinsic cardiac neural circuits, and measurable dynamic markers such as heart-rate variability (HRV) and baroreflex sensitivity [2,6,16].

### 1.2. Importance in Health and Disease and Scope of the Review

Changes in cardiac or neural health can cause neurocardiac comorbid disease features that reflect a bi-directional relationship. Neurologic insults, such as ischemic stroke, can trigger cardiac dysfunction through autonomic dysregulation and inflammation, resulting in a low HRV. Conversely, atrial fibrillation, for instance, significantly increases the risk of ischemic stroke through thromboembolism. This narrative review explores the heart–brain axis through an anatomical and functional lens assessing pathophysiological relationships that elucidates the clinical implications and unveils existing gaps for future research.

### 1.3. Literature Search Strategy

To ensure a comprehensive and objective synthesis of the literature, a structured search strategy was deployed across three primary electronic databases: PubMed/MEDLINE, Embase, and Scopus. The search encompassed peer-reviewed articles published from database inception through May 2026. The search queries were constructed using combinations of Medical Subject Headings (MeSH) terms and relevant text keywords, including but not limited to the following: “neurocardiology” OR “heart–brain axis” OR “neurocardiac”; “sympathetic hyperactivity” OR “catecholamine toxicity” OR “autonomic imbalance”; “cerebral blood flow” OR “cerebral perfusion” OR “fractional brain flow”; “neuroinflammation” OR “blood–brain barrier disruption” OR “microglial activation”; “Takotsubo cardiomyopathy” OR “cardioembolic stroke” OR “heart failure”. The inclusion criteria targeted randomized controlled trials, prospective or retrospective longitudinal cohort studies, animal models evaluating mechanistic neurocardiac pathways, and reviews. Studies were excluded if they lacked English full-text availability, or did not investigate a bidirectional physiological or pathophysiological relationship between the central nervous system and the cardiovascular system. To enhance the screening efficiency and ensure a full coverage of the emerging literature, the computational screening tool Elicit was utilized during the initial title and abstract screening phase to identify the relevant literature.

## 2. Anatomical and Functional Basis

### 2.1. Neural Pathways: Vagus Nerve, Sympathetic and Parasympathetic Innervation, and Neuro-Cardiac Junctions

#### 2.1.1. Parasympathetic (Vagal) Outflow

Parasympathetic preganglionic neurons originate in the dorsal motor nucleus and nucleus ambiguus in the brainstem, travel as the vagus nerve (CNX), and synapse on intracardiac ganglia within the heart [17]. The postganglionic fibers are short and release acetylcholine onto muscarinic receptors, which slows SA node firing and AV nodal conduction—slowing and stabilizing the heart rate [17]. The vagus is dominant at rest; it provides beat-to-beat fine control that is visible as HRV, with high-frequency (HF) HRV components directly modulating the vagal tone [17]. Multiple studies report that the vagus nerve is predominantly comprised of afferent (heart-to-brain) over efferent (brain-to-heart) fibers. For instance, Ottaviani et al. reports that 80% of vagal fibers are afferent [18], and Shaffer et al. reports vagal fibers to be 85–90% afferent [19]. This asymmetry demonstrates that the heart is not merely a passive recipient of central commands, but a major source of information flowing to the brain. This relationship elucidates an important pathophysiological relationship that is explored in this review.

#### 2.1.2. Sympathetic Outflow

The sympathetic outflow from the brain originates from sympathetic preganglionic neurons in the thoracolumbar spinal cord (T1–L2) and synapse in the stellate and paravertebral ganglia, from which postganglionic fibers reach the heart and travel along the coronary vessels [20]. These neurons secrete norepinephrine, which acts on β1-adrenergic receptors to increase the heart rate (chronotropy), conduction velocity through the AV node (dromotropy), and contractile force (inotropy) [21,22]. The sympathetic system is the heart’s accelerator: it is activated by stress, exercise, hemorrhage, and emotion, raising the cardiac output to meet the increased metabolic demand [21,22]. The primary regulator of the sympathetic tone at the circuit level is the rostral ventrolateral medulla (RVLM); the bilateral lesioning or pharmacologic blockade of the RVLM results in decreased sympathetic activity [23]. An important concept of downstream physiology is that sympathetic cardiac effects are not independent of the background vagal state: the sympathetic heart-rate effects are substantially smaller when the vagal tone is high, and the changes in cardiac activity attributed to sympathetic control is concomitantly controlled based on the vagal tone [24].

#### 2.1.3. Neuro-Cardiac Junctions, Intrinsic Cardiac Neural Network Organization, and Local Neuron–Cardiomyocyte Communication

Neuro-cardiac junctions are specialized contact sites between intrinsic cardiac neurons and cardiomyocytes characterized by distinct structural and molecular features that enable bidirectional communication. Structurally, these junctions consist of stable intercellular contacts with accumulated dystrophin on cardiomyocyte membranes [25], the preferential localization of NGF receptors (TrkA) [25,26], and enriched β-adrenergic signaling components [27]. The junctions create insulated microdomains maintaining neurotrophic factor concentrations approximately 1000-fold higher than ambient levels [25], enabling efficient signaling despite the low background concentrations. Regional heterogeneity exists within the junctions, with epicardial cardiomyocytes receiving more neuronal contacts than endocardial cells [27] and the local innervation density directly correlating with the cardiomyocyte size across mammalian species [27].

Functionally, neuro-cardiac junctions facilitate both anterograde neurotransmission (via norepinephrine and acetylcholine activating adrenergic and muscarinic receptors) [27] and retrograde neurotrophic signaling (cardiomyocytes providing NGF to sustain neuronal viability) [26]. This bidirectional communication operates through confined subcellular signaling domains that enable the rapid modulation of cardiac automaticity [28], conduction [28], and long-term structural remodeling through the control of cardiomyocyte proteolysis [27]. Intrinsic cardiac neurons serve as the final integration site for neural control, mediating local reflex responses and integrating parasympathetic and sympathetic inputs through interconnected ganglia with synaptic convergence [28]. The disruption of the junction structure, such as the loss of dystrophin, reduces innervation by 74% and impairs signaling, demonstrating the essential structure–function coupling [25]. The temporal dynamics distinguish acute electrophysiological effects occurring within milliseconds from chronic trophic influences regulating the myocardial architecture over days to weeks [27].

### 2.2. Circulatory Connections: Cerebral Perfusion and Cardiac Output

Cerebral perfusion is functionally linked to cardiac output through direct hemodynamic flow delivery, cerebral autoregulation, autonomic nervous system–mediated cardiovascular adjustments, and intracranial oxygen-sensing pathways [29]. The brain normally receives about 15% of the cardiac output and maintains a stable blood flow across a wide pressure range in young healthy individuals but becomes increasingly dependent on the cardiac output with aging, a reduced autoregulatory reserve, and cardiovascular or endothelial dysfunction [29].

#### 2.2.1. Direct Hemodynamic Coupling Between Cardiac Output and Cerebral Blood Flow

In cerebrovascular physiology, cerebral blood flow (CBF) is primarily regulated by metabolic demand, carbon dioxide arterial tension, and cerebral autoregulation maintaining constant flow across varying mean arterial pressures (MAPs). However, the current work of literature explores whether cardiac output exerts a direct, independent influence on CBF beyond its effect on MAP. The evidence is divided but leans toward affirming an independent cardiac output effect, particularly under specific physiological conditions. Several studies demonstrated a linear relationship between the cardiac output and CBF velocity. Ogoh et al. showed that middle cerebral artery (MCA) mean blood velocity (MCA Vmean) and cardiac output were linearly related both at rest (*p* < 0.001) and during exercise (*p* = 0.035) [30]. Lie et al., using a combined model of reduced preload and increased afterload in 16 healthy volunteers, found that both MAP and cardiac output had independent effects on MCA velocity: a change of 1 L/min in cardiac output was associated with a 3.41 cm/s change in MCA velocity (95% CI 2.82–4.00, *p* < 0.001), comparable to the 3.11 cm/s effect of a 10 mmHg change in MAP (95% CI 2.51–3.71, *p* < 0.001) [31]. Furthermore, there was a statistically significant interaction between MAP and cardiac output on internal carotid artery velocity and cerebral oxygen saturation [31]. Meng et al., in their review, summarized that alterations in the cardiac output contributes to changes in CBF independent of other CBF-regulating parameters including blood pressure and carbon dioxide [32].

In contrast, some studies report no direct effect of cardiac output on cerebral perfusion. Deegan et al. demonstrated that cardiac output responses did not correlate with CBF autoregulatory indices during thigh-cuff-induced hypotension, concluding that the cardiac output does not affect the dynamic cerebral autoregulatory response to sudden hypotension [33]. Henriksen et al. similarly found no effects of the cardiac output or cardiac index on CBF in 31 healthy older adults [34]. In premature neonates, Victor et al. found no apparent relationship between the left or right ventricular output and indicators of cerebral perfusion, provided that the mean blood pressure exceeded 30 mmHg [35]. Davis and Sundt provided nuanced results in an animal study: isoproterenol, nonselective β-adrenergic agonist increased the cardiac output by 38–72% without changing CBF, demonstrating the brain’s ability to resist increases via autoregulation [36]. However, moderate hypovolemia that did not change the mean arterial blood pressure nevertheless significantly decreased both the cardiac output and CBF, revealing a vulnerability that could not be attributed to changes in the perfusion pressure alone [36]. The contrasting evidence does seem to suggest that CBF is not solely passive to MAP. Instead, the cardiac output seems to exert a distinct, independent influence that becomes highly pronounced during hypovolemia and physiological stress. While cerebral autoregulation can effectively shield the brain from increases in systemic flow (surges in cardiac output), it may be uniquely vulnerable to decreases in systemic flow (drops in cardiac output), even when systemic compensatory mechanisms manage to keep the blood pressure stable.

#### 2.2.2. Cerebral Autoregulation as a Buffering Mechanism

Cerebral autoregulation is the primary mechanism by which the brain maintains a relatively constant blood flow across a range of perfusion pressures. Autoregulation maintains a relatively stable CBF with slow blood pressure changes between approximately −20 and +20 mmHg, with a baseline CBF of approximately 50 mL/100 g per min and ischemic thresholds below approximately 22 mL/100 g per min [29]. Autoregulation can operate statically or dynamically depending on whether steady-state hemodynamic changes occur or whether rapid perturbations occur, respectively [35]. Importantly, cerebral autoregulation does not respond effectively to rapid changes in MAP, explaining the transient falls in cerebral blood velocity on standing and associated light-headedness [37]. Van Lieshout et al. characterized autoregulation as a frequency-dependent phenomenon with the attenuated transfer of MAP oscillations to cerebral blood velocity at low frequencies [37]. Vascular reactivity to CO_2_ is a potent modulator of cerebral vessel diameter through pH changes [36,38]. Notably, propranolol, a non-selective β-antagonist, abolished the cardiac output response to elevated PaCO_2_ but not the CBF response, dissociating the CO_2_-mediated cerebral vascular reactivity from cardiac output changes [36]. Furthermore, the autonomic nervous system contributes to both static and dynamic autoregulation through sympathetic vasoconstriction, which serves a protective role against blood–brain barrier (BBB) disruption during hypertension and high-cardiac-output states [39,40]. Altogether, the evidence suggests that cerebral autoregulation acts as a buffering mechanism to protect the brain hemodynamic volatility.

#### 2.2.3. Fractional Brain Flow and Cardiac Output Distribution

Several studies have examined the fraction of the cardiac output distributed to the brain and how these change with age, sex, and physiological state. A widely accepted baseline is that approximately 15–20% of the cardiac output is received by the brain in healthy adults at rest [39,41,42]. However, this fraction is not static. Xing et al. measured the CBF/cardiac output ratio index in 139 subjects aged 21–80 years and found that it decreased by 1.3% per decade, driven by a decreasing CBF while the cardiac output remained unchanged [41]. Women had a higher CBF, lower cardiac output, and, thus, a higher CBF/CO ratio index than men across their lifespan [41]. Henriksen et al. reported similar sex differences: the fractional brain flow was 8.6% in males versus 12.5% in females (*p* = 0.003) and was inversely correlated with the cardiac index (r^2^ = 0.22, *p* = 0.008) [34]. The fractional brain flow may also serve as a useful marker of adequate brain perfusion in the context of aging and cardiovascular disease [34]. The “selfish brain hypothesis,” as discussed by Lie et al., suggests that the brain prioritizes its own blood flow by adjusting the extracerebral vascular resistance in response to cardiac output reductions, such that CBF constitutes a higher proportion of the cardiac output as the total cardiac output is reduced [31]. This concept aligns with the framework proposed by McBryde et al., who speculated that local cerebral oxygen tension is a major determinant of the mean level of arterial pressure, potentially leading to systemic hypertension to preserve brain perfusion [39]. Overall, the distribution of the cardiac output to the brain is a highly regulated, plastic variable governed by age, sex, and compensatory physiology.

#### 2.2.4. Age-Related Changes in the Cardiac–Cerebral Relationship

A consistent theme is that the cardiac output–cerebral perfusion relationship is modified by aging. Bronzwaer et al. compared healthy young (19–27 years), middle-aged (51–61 years), and elderly (70–79 years) subjects and found that a linear relationship between cardiac output changes and MCA velocity was present in middle-aged (*p* < 0.01) and elderly (*p* = 0.04) subjects but not in young subjects (*p* = 0.45), after accounting for concurrent changes in MAP and end-tidal CO_2_ [43]. This implies that, with aging, brain perfusion becomes increasingly dependent on cardiac output [43]. Siennicki-Lantz and Elmståhl, studying 341 older adults aged 73–87 years, found that cardiac output was significantly associated with regional CBF across many brain areas, with the strongest associations in the posterior and cerebellar areas as well as border zone/watershed regions, independently of central or peripheral arterial stiffness [42]. The decline in CBF with aging was reported at approximately 0.38–0.45% per year [42]. Jefferson et al. found that a lower cardiac index corresponded to a lower resting CBF specifically in the temporal lobes (left: *β* = 2.4, *p* = 0.001; right: *β* = 2.5, *p* = 0.001) in 314 older adults (mean age 73 years), independently of prevalent cardiovascular disease [44]. Interestingly, this association was present only in cognitively normal participants and not in those with mild cognitive impairment (CI) [44]. These findings suggest that normal aging comprises the progressive failure of cerebral autoregulation, leaving the brain’s perfusion to be significantly reliant on cardiac efficiency. This systemic–cerebral dependency may result in a predictable annual decline in CBF that preferentially targets vulnerable watershed zones and temporal lobes. Interestingly, the disappearance of this cardiac–cerebral link in individuals with mild cognitive impairment suggests a pathophysiological tipping point. In healthy older adults, maintaining a robust cardiac output is critical to protecting brain perfusion; however, once cognitive impairment manifests, the underlying neurodegenerative or localized vascular pathology may decouple the brain from the systemic cardiac influence entirely.

#### 2.2.5. Exercise as a Window into Cardiac–Cerebral Coupling

Exercise provides a dynamic physiological challenge that reveals the interplay between cardiac output and cerebral perfusion. During exercise, CBF likely increases through multiple modulating variables that interact [38]. Ogoh et al. showed that, while the MCA Vmean–cardiac output relationship was linear during both rest and exercise, the slope was steeper at rest (*p* = 0.035), suggesting that the exercise-induced sympathoexcitation and redistribution of blood between cerebral and systemic circulations modify the relationship [30]. Ide et al. used metoprolol, a β1-adrenergic antagonist, to pharmacologically reduce the cardiac output during exercise and found that, during cycling, metoprolol reduced the increase in cardiac output (222 ± 13% vs. 260 ± 16%) and the increase in MCA velocity (59 ± 3 to 66 ± 3 vs. 60 ± 2 to 72 ± 3 cm/s; *p* < 0.05) [45]. This effect was observed during large-muscle-mass exercise (cycling) but not during small-muscle-mass exercise (rhythmic handgrip) [45], suggesting that the cardiac output limitation becomes relevant when the systemic demand is high. Later, Querido and Sheel noted that the impairment of the cardiac output increase during exercise with a large muscle mass similarly impairs the increase in CBF velocity, reinforcing cardiac output as a key determinant of the CBF response to exercise [38]. Koike et al. demonstrated the clinical relevance of this relationship: patients with valvular heart disease showed significantly lower increases in cerebral oxyhemoglobin during exercise than normal subjects, with 15 of 33 patients exhibiting a significant decrease in cerebral oxygenation [46]. These findings reveal the hierarchical physiological triage. During strenuous, large-muscle exercise, the brain’s perfusion becomes strictly tethered to the heart’s pumping capacity. If the cardiac output is limited, the systemic demand overrides typical cerebral preservation mechanisms, resulting in a measurable deficit in brain perfusion and oxygenation.

### 2.3. Heart–Brain Feedback Loops

Heart–brain feedback loops are bidirectional communication circuits in which the brain modulates the cardiac function via sympathetic and parasympathetic efferent pathways, while the heart simultaneously transmits mechanical and chemical information back to the brain—predominantly through vagal afferent fibers—creating a closed-loop system that regulates the heart rate, blood pressure, cognition, and emotional processing, with HRV serving as the principal biomarker of this system’s integrity.

There exists a hierarchical architecture within this system in which the medulla serves as a primary integration hub, receiving descending inputs from cortical and subcortical regions (the prefrontal cortex, anterior cingulate, insula, and amygdala) and ascending inputs from the heart via vagal and spinal afferents [19,47,48]. The nucleus of the solitary tract (NST) within the medulla acts as a critical relay station that modulates the sympathetic and parasympathetic outflow [19]. The intrinsic cardiac nervous system, or the “little brain of the heart”, then emerges as an autonomous processing center that integrates mechanosensory and chemosensory information locally before relaying signals centrally [19,48,49].

The autonomic nervous system provides the primary fast-acting communication channel, with the parasympathetic (vagal) and sympathetic branches exerting opposing influences on cardiac chronotropy [19,47,48]. Beyond classical neurotransmission, the heart–brain axis involves humoral signaling, including the cardiac endocrine function via the atrial natriuretic peptide (ANP) [19], and immune-mediated communication through pro-inflammatory cytokines and microRNA-carrying extracellular vesicles [47]. Efferent signaling from brain to heart involves both sympathetic acceleration (norepinephrine at cardiac β-adrenergic receptors) and parasympathetic deceleration (acetylcholine at muscarinic receptors on the SA node) [47,48]. Triggers for efferent modulation include mental stress and emotion [50]. Afferent signals from the heart convey mechanical (pressure and stretch) and chemical information to the brain, influencing the activity in the subcortical and frontocortical areas [19,48]. These afferent inputs modulate not only autonomic reflexes (e.g., baroreflex) but also higher cognitive and emotional processing [19].

**Figure 1:**
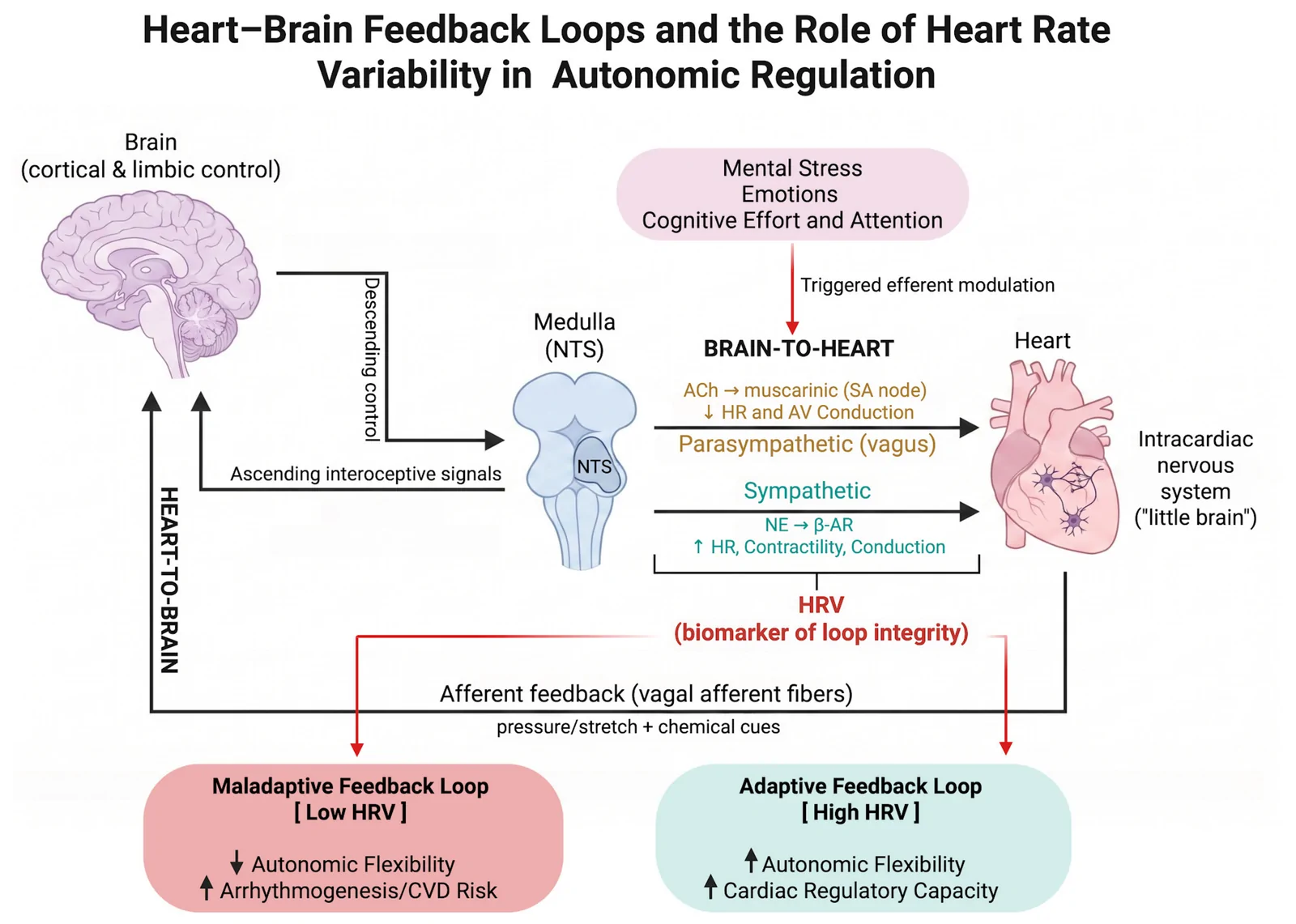
Figure depicting heart–brain feedback loops regulating cardiovascular, cognitive, and emotional function. The heart–brain axis operates as a bidirectional communication network in which higher cortical and limbic regions regulate cardiac activity through descending autonomic pathways, while the heart continuously transmits physiological information back to the brain via ascending afferent pathways. Emotions, mental stress, and cognitive demands modulate autonomic output through the medulla, particularly the NTS, resulting in parasympathetic (vagal) and sympathetic regulation of cardiac function. Parasympathetic signaling, mediated by Ach acting on muscarinic receptors, reduces heart rate and atrioventricular conduction, whereas sympathetic signaling, mediated by NE acting on β-adrenergic receptors (β-AR), increases heart rate, myocardial contractility, and conduction. The intrinsic cardiac nervous system (“little brain of the heart”) integrates local neural and sensory information and contributes to cardiac regulation. Simultaneously, mechanical and chemical signals generated by the heart are conveyed to the brain primarily through vagal afferent fibers, forming a closed-loop feedback system. HRV serves as a key biomarker of heart–brain axis integrity, reflecting the dynamic balance between sympathetic and parasympathetic activity. High HRV indicates adaptive autonomic flexibility and low HRV indicates maladaptive regulation and greater cardiovascular risk. Ach, acetylcholine; AV, atrioventricular; β-AR, β-adrenergic receptor; HR, heart rate; HRV, heart rate variability; NE, norepinephrine; NTS, nucleus tractus solitarius. Created in BioRender. Singh, A. (2026) https://BioRender.com/i5byhe9.

The functional effects of heart–brain feedback loops extend well beyond cardiac rate control. Cognitive and emotional domains are consistently linked to cardiac autonomic regulation through the neurovisceral integration framework: individuals with a higher resting HRV demonstrate superior executive function and more flexible emotional responses, while those with a low HRV exhibit cognitive inflexibility and impaired prefrontal inhibitory control (Figure 1) [51]. The maladaptive end of this spectrum is exemplified by perseverative cognition (e.g., worry and rumination), which produces a sustained autonomic activation that increases the cardiovascular risk over time [51]. At the cardiac level, disrupted feedback loops contribute to arrhythmogenesis [50].

When these loops function optimally (high HRV), they promote physiological resilience and cognitive flexibility. Conversely, chronic disruptions trap the system in a maladaptive, low-HRV feedback loop. Over time, this sustained autonomic activation transitions from a psychological burden to a physical pathology, directly contributing to clinical conditions. Understanding the granularity of these circuits is key for targeted, holistic psychological and cardiological care.

## 3. Mechanisms of Pathophysiological Axis

### 3.1. Brain-to-Heart Pathways

#### 3.1.1. Acute Brain Injury and Neurogenic Cardiac Injury

**Figure 2:**
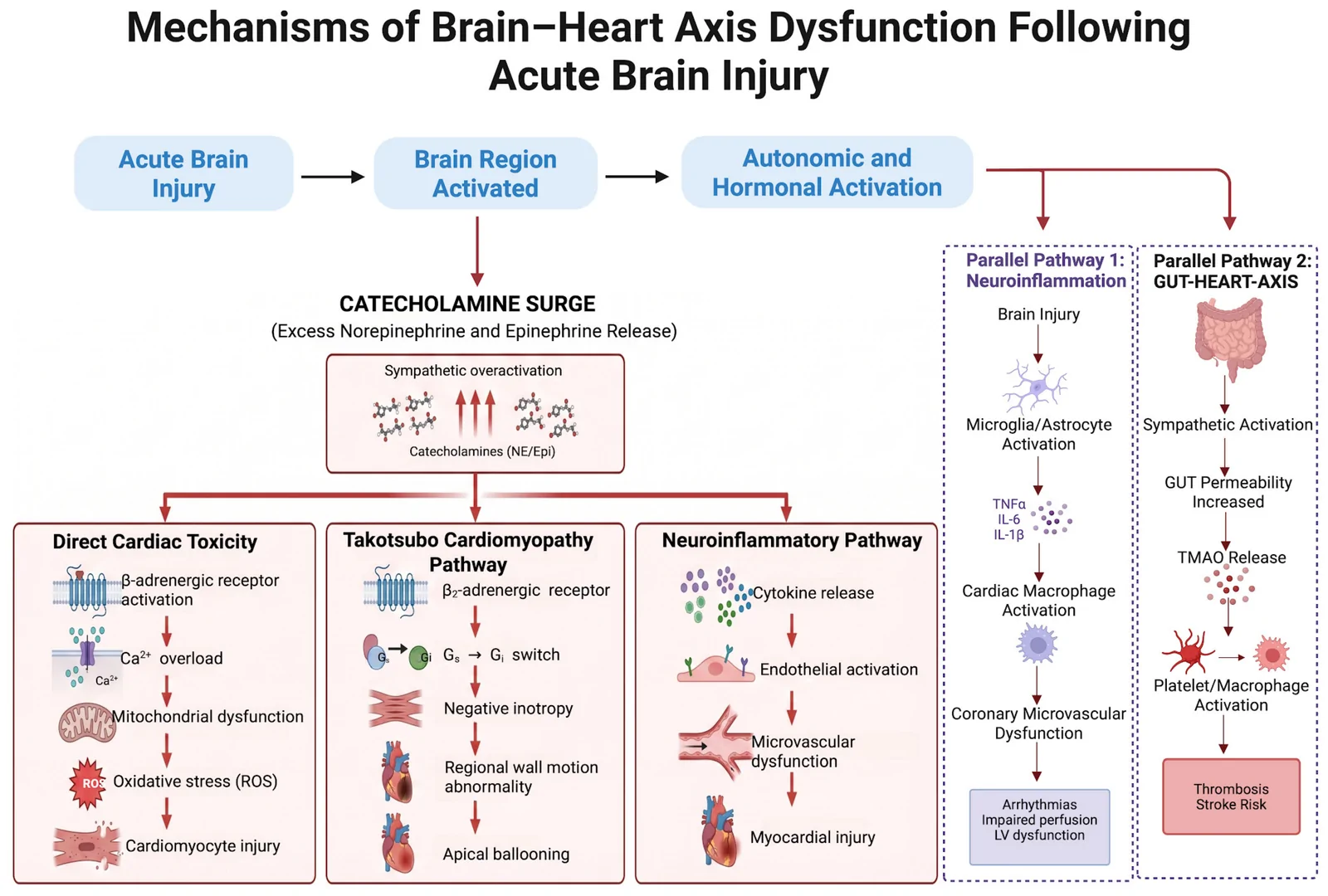
Mechanisms of brain–heart axis dysfunction following acute brain injury. Acute brain injury activates autonomic and hormonal stress pathways, leading to a catecholamine surge with excess norepinephrine and epinephrine release. The ensuing catecholamine surge contributes to direct cardiac toxicity through β-adrenergic receptor activation, calcium overload, mitochondrial dysfunction, and ROS, leading to cardiomycyte injury. Catecholamine-mediated β2-adrenergic receptor signaling may also induce a Gs-to-Gi switch, producing negative inotropy, LV hypokinesis, and Takotsubo cardiomyopathy. In parallel, neuroinflammatory pathways promote cytokine release, endothelial activation, microvascular dysfunction, and myocardial injury, contributing to CVDs. Additional inflammatory and sympathetic signaling activate microglia, astrocytes, and cardiac macrophages, resulting in coronary microvascular dysfunction, impaired myocardial perfusion, and arrhythmogenesis. Furthermore, sympathetic activation can disrupt gut barrier integrity, increase TMAO production, and stimulate platelet and macrophage activation, thereby enhancing thrombosis and stroke risk. Collectively, these interconnected pathways illustrate the multifaceted mechanisms through which acute brain injury induces cardiovascular dysfunction. CVDs, cardiovascular diseases; NE, norepinephrine; Epi, epinephrine; ROS, reactive oxygen species; TMAO, trimethylamine-N-oxide; LV, left ventricular. Created in BioRender. Singh, A. (2026) https://BioRender.com/nyfe9h2.

Acute brain injury describes a variety of pathologies and encompasses damage to the brain from events such as ischemic stroke, spontaneous subarachnoid hemorrhage, spontaneous intracerebral hemorrhage, and traumatic brain injury. According to Coppalini et al., following injury to the brain, there is potential for a surge of catecholamines to be released, in addition to a systemic inflammatory response [52]. In cases of traumatic brain injury, 25–35% of patients will have cardiac complications, such as supraventricular arrhythmias (five-to-ten-fold risk compared to the general population) [52]. Other examples of cardiac dysfunction from brain injury include Takotsubo cardiomyopathy, regional wall motion alteration, troponin elevation, and myocardial stunning [52]. Lele et al. found that patients with ischemic stroke were found to have similar cardiac manifestations: 61% had a prolonged QTc and 12% were found to have a reduced ejection fraction [53]. Additionally, acute brain injury with the presence of elevated Brain Natriuretic Peptide (BNP) was associated with an increased risk of brain death and comfort care transitions, while acute brain injury with elevated troponin saw an increased risk of death, brain death, and transition to comfort care (Figure 2) [53].

Other studies focus on associations between specific types of brain injury and cardiac manifestations. Macmillan et al. found that subarachnoid hemorrhage induced contraction band necrosis and focal cell death in the heart, which may serve as a pathophysiological mechanism for wall motion abnormalities or cardiogenic shock which may result post insult [54]. Gobeske et al. described the occurrence of medulla oblongata hemorrhage, in which a cardiac workup with an ECG, echocardiogram, chest X-ray, and troponin was done. Findings included an enlarged heart, reduced ejection fraction, elevated troponin, and basal hypokinesis of the heart, indicating the diagnosis of reverse Takotsubo cardiomyopathy [55]. The authors noted that this is an unusual case because Takotsubo cardiomyopathy is typically caused by larger bleeding in areas other than the brainstem; however, the cardiac influence of areas within the medulla oblongata such as the nucleus tractus solitarius and ventrolateral medulla help delineate the findings of this case [55]. These studies highlight the importance of including a cardiac workup following acute brain injuries, considering the highly interconnected nature of the heart and brain.

#### 3.1.2. Stress-Induced Cardiomyopathy (Takotsubo)

Takotsubo cardiomyopathy is a transient weakening of the heart characterized by hypokinesis of the left ventricle and regional wall motion abnormalities. Although generally recognized as a transient condition, Takotsubo cardiomyopathy carries a 4–5% mortality rate, and, in some cases, can lead to permanent damage to the heart [56]. This condition typically follows extreme physical and, in some cases, emotional, stress to the brain. Examples of such stress include stroke, traumatic brain injury, epilepsy, or subarachnoid hemorrhage [52]. According to Gopinath et al. and Gobeske et al., insult to the brain, particularly in areas such as the insular cortex, anterior cingulate gyrus, amygdala, brainstem, or hypothalamus, can induce surges of catecholamines to be released into circulation for long periods of time (Figure 2) [55,57]. This surge of catecholamines promotes the ligand-mediated trafficking of Gs beta-2 receptors to inhibitory Gi beta-2 receptors [57]. This causes catecholamines to take on a negative inotropic effect, which delineates the hypokinesis characteristic of Takotsubo cardiomyopathy [57]. However, other proposed mechanisms that may be involved in this condition are the inhibition of myocardial survival pathways, plaque rupture/thrombosis, coronary vasospasm, and microcirculatory dysfunction [56].

According to Pelliccia et al., the risk factors for Takotsubo cardiomyopathy include menopause in females, in which the estrogen reduction likely promotes endothelial dysfunction [58]. Fu et al. further characterized this relationship and found that interactions with G-Protein Coupled Estrogen Receptors (GPERs) and estrogen prevented the internalization of Beta-2 Adrenergic Receptors and limited the Gi activity of these receptors in the presence of epinephrine. This interaction was also associated with lower BNP and Lactate Dehydrogenase levels, suggesting less severe myocardial injury. When GPER antagonists (G15) or the shRNA knockout of GPER were induced, these markers of cardiac disease were reversed, characterizing a cardioprotective role of estrogen [59]. Additional risk factors and predictors of mortality include non-epicardial coronary ischemia, left ventricular outflow tract obstructions, a high Simplified Acute Physiology II Score, renal impairment, malnutrition, norepinephrine infusion, and thrombocytopenia [60]. The diagnostic criteria are not universally agreed upon for Takotsubo cardiomyopathy, although some general commonalities are proposed within the literature. These include the transient hypokinesis, akinesis, or dyskinesis of the left ventricular midsegments (with or without apical involvement); regional wall motion abnormalities that extend beyond one epicardial vascular distribution; a stressful trigger; the absence of obstructive coronary artery disease and evidence of plaque rupture; new ST-segment elevation and/or T-wave inversion on ECG; a modest elevation in cardiac troponin; and the absence of pheochromocytoma and myocarditis [57].

Takotsubo cardiomyopathy represents a profound failure of the body’s emergency adaptation system. The very mechanism designed to shield the heart muscle cells from a massive adrenaline surge (Gi receptor trafficking) creates an acute, life-threatening structural phenomenon.

#### 3.1.3. Sympathetic Overactivation and Catecholamine Toxicity

There are a variety of pathologies within the brain that can harmfully modify cardiac functionality, including sympathetic overactivation and neuroinflammation. Brain injury can lead to the formation of damage-activating molecular patterns, which activate microglia and astrocytes to prefer the pro-inflammatory M1 types (Figure 2) [61]. This promotes the release of cytokines which can activate the Hypothalamic–Pituitary–Adrenal (HPA) axis, disrupt the BBB, impair autonomic control in the heart, and promote the development of cardioembolic stroke or chronic cerebral hypoperfusion [61]. Sympathetic overactivation is another mechanism in which stress or injury to the brain can impact cardiovascular health from a variety of pathways. For example, a surge of catecholamines from sympathetic overactivation can disrupt atherosclerotic plaques, significantly increasing the risk of vascular issues, arrhythmias, or stroke [61]. Sympathetic overactivation also modulates the inflammatory response through the reduction of Interleukins (IL)-1, 2, and 6 and Tumor Necrosis Factor (TNF)-α. These modulatory changes remove the inhibitory mechanisms of immune activation, promoting inflammatory environments. Such overactivation can lead to the activation of cardiac macrophages, for example. These macrophages can infiltrate the myocardium and stimulate microvascular endothelial cells, leading to coronary microvascular dysfunction, impaired myocardial perfusion, left ventricle dysfunction, and the development of arrhythmias such as AF [61]. Furthermore, the gut can be involved in the heart–brain axis especially in the context of sympathetic overactivation; catecholamines can increase gut permeability and reduce motility, and toxins from the microbiome have been found to more readily enter circulation. For instance, microbiota-produced trimethylamine-N-oxide can further activate resident macrophages and platelets, leading to an increased risk of thrombosis (Figure 2) [61].

Additionally, neurogenic causes of sympathetic overactivation can also lead to deleterious complications such as sudden death [62]. This is especially relevant for insults including stroke, epilepsy, brain trauma, and drug overdose. Interestingly, right-sided ventromedial prefrontal cortex lesions have been demonstrated to be associated with cardiac injury [62]. Patients with a history of traumatic brain injury are at an increased risk of sudden death even if the TBI occurred years before [62]. These characterizations of cardiac injury following brain injury and sympathetic overactivation highlight the importance of robust diagnostic criteria and workup that covers both heart and brain function [63].

### 3.2. Heart-to-Brain Pathways

**Figure 3:**
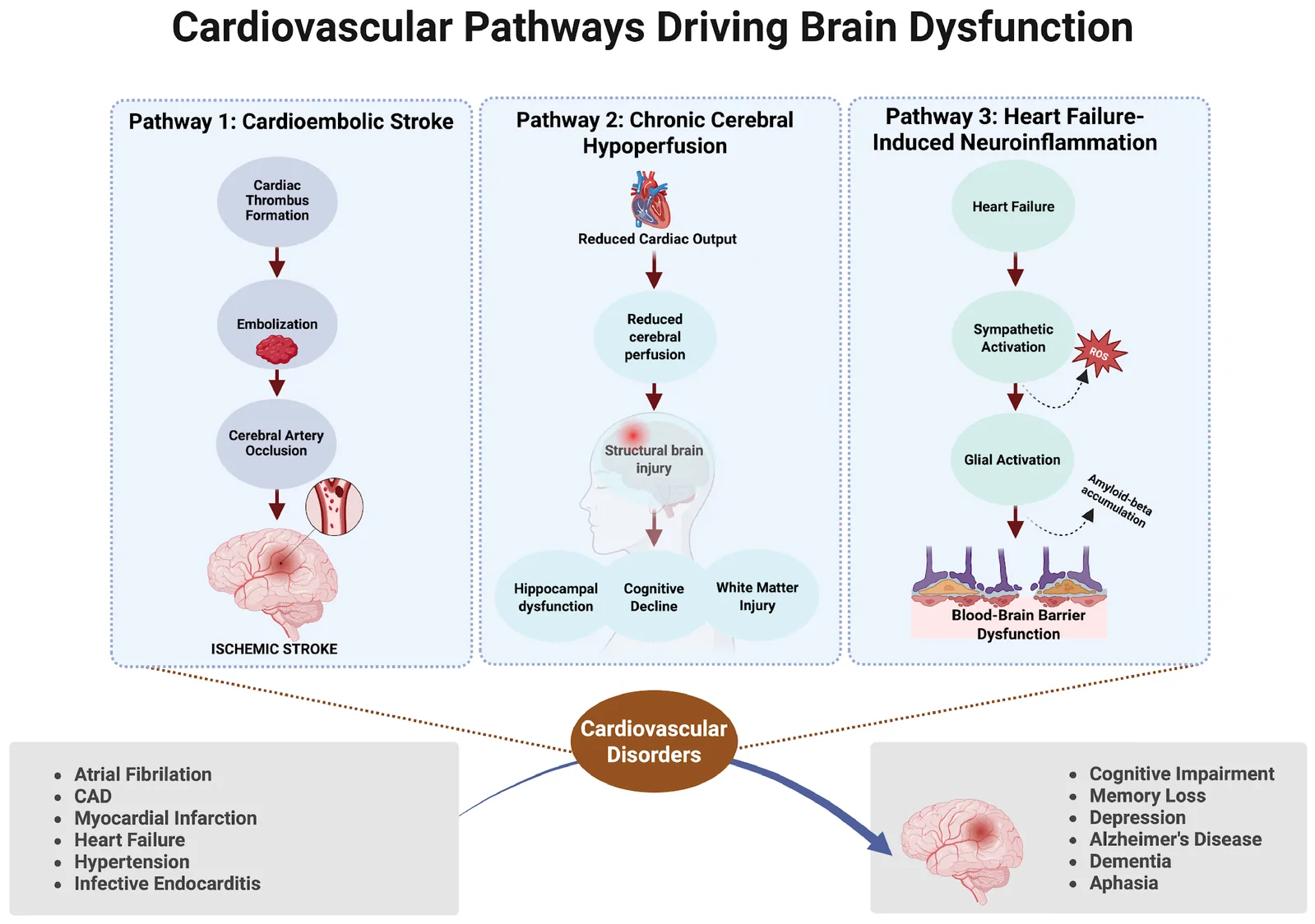
Cardiovascular pathways driving brain dysfunction. Schematic summary of major cardiovascular mechanisms contributing to neurological injury. Cardioembolic events, commonly associated with atrial fibrillation or other cardiac disorders, can promote thrombus formation, embolization, cerebral artery occlusion, and ischemic stroke. Chronic reductions in cardiac output may cause cerebral hypoperfusion, structural brain injury, hippocampal dysfunction, cognitive decline, and white matter injury. Heart failure may further contribute to brain dysfunction through sympathetic activation, oxidative stress, glial activation, amyloid-β accumulation, and blood–brain barrier disruption. Together, these pathways link cardiovascular disorders with cognitive impairment, memory loss, dementia, Alzheimer’s disease, depression, and other neurological outcomes. ROS, reactive oxygen species. Created in BioRender. Singh, A. (2026) https://BioRender.com/btuoq4k.

The major mechanisms linking cardiovascular disease to neurological dysfunction through the heart-to-brain axis are summarized in Figure 3.

#### 3.2.1. Cardioembolic Stroke

Stroke is the leading cause of disability and the third leading cause of death in developed countries, in which 15–30% of strokes are cardioembolic in origin [64]. Various pathologies can occur in the heart and vessels to promote the formation of a stroke: AF can lead to thrombi that travel to the brain; hypertension can lead to issues in both the heart and brain in the forms of left ventricular hypertrophy and intracerebral hemorrhage, respectively; infective endocarditis in the heart can promote the formation of thrombi; valvular diseases such as mitral stenosis or the presence of mechanical heart valves can promote thrombosis; and HF with a reduced ejection fraction can lead to left ventricular dilation with cardiac remodeling, increasing the risk of cardioembolic stroke [65]. Interestingly, Vemmos et al. have also investigated the consequences of cardioembolic stroke on cardiovascular parameters: for example, cardioembolic stroke with the additional risk factors of a history of hypertension, high stroke severity, hemorrhagic transformation, and brain edema were associated with higher 24-h blood pressure [66]. Additionally, cardioembolic stroke with a history of HF was found to be associated with lower 24-h blood pressure [66]. Martin et al. have also noted associations with myocardial infarction and cardioembolic stroke [67].

In the study by Martín et al., patients with a first stroke at least 3 months following a myocardial infarction underwent brain computed tomography (CT), extra/transcranial Doppler ultrasound, 12-lead electrocardiogram, and transthoracic echocardiography [67]. They found that all patients had akinetic left ventricles, while only 12% had visible thrombi and 12% had hypertension [67]. Additionally, severe carotid artery occlusion (defined as 50% or more stenosis or occlusion) was found in the ipsilateral artery to brain infarcts in 21% of patients with anterior circulation stroke [67]. Moreover, 15% of patients had additional cardiac manifestations in addition to akinetic left ventricles, in which AF was the most common. Each of these findings may mediate the relationship between myocardial infarction and cardioembolic stroke, in addition to risk factors that were found to independently increase the risk of stroke (hypercholesterolemia, older age, male sex, and vascular claudication) [67].

The literature also highlights some molecular mechanisms governing cardioembolic stroke in addition to its risk factors [68]. Shi et al. found that higher levels of Adenosine Phosphoribosyltransferase (APRT) and high levels of CD40L were each protective against cardioembolic stroke, whereas higher levels of Interleukin-15 Receptor Subunit Alpha (IL15RA) were associated with a higher risk of cardioembolic stroke (Figure 3). There are many direct and indirect effects that these molecules have on stroke risk: 17.5% of the effect of CD40L was from reducing the risk of AF, which concurrently lowers the stroke risk [68]. Importantly, these molecular targets may be the focus of future research for therapeutics. Shi et al. also investigated the efficacy of compounds from Panax Notoginseng, an herbal medicine, such as ginsenoside Rg1, which were found to alter the expression of IL15RA and APRT. Although more work is needed, there exists promise for these compounds to act as prophylactic therapeutics for individuals with an increased suspected risk of cardioembolic stroke [68].

Many studies in the literature stress the importance of the appropriate diagnosis and workup of stroke as it relates to cardiovascular health and the identification of its origin [64,65]. Even after the initial workup, it is estimated that ~30% of stroke cases involve undetermined origins [64]. Suspicion of the cardiac origin of a stroke is built from a variety of domains and signs including clinical (sudden onset of maximal deficit, and rapid regression of symptoms); magnetic resonance imaging (MRI) and CT imaging (hemorrhagic transformation, and hyperdense artery sign in the absence of arterial pathology); ultrasound imaging (occlusion of the carotid artery by a mobile thrombus, and early recanalization of an arterial occlusion); and laboratory evaluation (elevated troponin, and elevated brain natriuretic peptide) [64]. Certain deficits such as Wernicke’s aphasia, global aphasia without hemiparesis, Wallenberg’s syndrome, cerebellar infarcts, and top-of-the-basilar artery syndrome are also associated with cardioembolic stroke specifically [64]. To further characterize the pathogenesis of stroke, clinicians can correlate with clinical signs such as fever (indicating infective endocarditis) or irregularly irregular pulses (indicating AF) [65]. Finally, certain systemic diseases should also be considered including conditions such as systemic lupus erythematosus and antiphospholipid antibody syndrome, which can be implicated in stroke [65].

#### 3.2.2. Chronic Cerebral Hypoperfusion and Cognitive Decline

Many studies have characterized the association between chronic cerebral hypoperfusion and cognitive decline through various mechanistic frameworks, most notably the structural–functional model of the heart–brain axis [69,70,71]. This model posits that systemic cardiovascular risk factors and structural cardiac changes translate directly into altered cerebral perfusion, driving macro- and microstructural neural degeneration that manifests as clinical cognitive deficits [69]. Within the framework, a variety of data has been collected on vascular risk factors, heart and brain MRI imaging, and cognitive test performance (as measured by fluid intelligence tasks, visual memory tasks, reaction time tasks, and prospective memory tasks) [69,70,71]. The measurements of deteriorations in cognitive functions in the presence of high vascular risk factors also typically demonstrated abnormalities in the heart and brain structures—namely, a lower myocardial intensity, lower grey matter volume, and poorer thalamic white matter integrity [69]. In another study, similar findings were noted—coronary artery disease was associated with grey matter atrophy, white matter structural changes, and functional network connectivity changes [70]. Here, the network changes included the default mode network of the brain being functionally impaired [70]. In addition, this study noted that neuroinflammation in the presence of coronary artery disease was mediated by factors such as C-Reactive Protein and Interleukin-6 [70]. Yet, other studies in rats have also examined brain tissue following the induction of chronic cerebral hypoperfusion, highlighting damages to the CA1 region of the hippocampus [70]. Additionally, chronic cerebral hypoperfusion was associated with increased acetylcholinesterase activity, which may act as a mechanism for the degradation of memory [70]. These structural and functional findings support the structural–functional model’s proposition that cardiovascular risk and damage can reduce blood flow to the brain, leading to cognitive decline (Figure 3) [69,70,71]. Comorbidities including hypertension, kidney disease, and diabetes mellitus may also exacerbate this relationship [70].

There are a variety of pathologies that can result in such decreased blood flow to the brain. One of the most common includes AF, which is a significant risk factor for cardioembolic stroke [72]. However, studies have also characterized an association between AF and cognitive decline even in the absence of stroke. Specifically, patients with AF experience a 1.4–2.2-fold higher risk of cognitive decline and dementia even when excluding cases of stroke [72]. Many mechanisms have been proposed for this association and highlight the complexity within pathologies of the heart–brain axis. For example, in addition to cardioembolic stroke, silent cerebral infarcts may take place in the context of AF. Additionally, there exists the possibility of small vessel disease, structural brain atrophy, oxidative stress, and neuroinflammation to act as mediators in the association between AF and cognitive decline (Figure 3) [70,72]. Most importantly to the structural–functional model of the heart–brain axis is chronic cerebral hypoperfusion; AF can reduce the cardiac output in such a way as to negatively influence the brain structure, thus leading to cognitive decline [70,72]. Studies investigating these relationships stress the need for the thorough follow-up, testing, and treatment of AF as it relates to neurologic consequences [72]. Direct Oral Anti-Coagulation (DOAC) mediations should also be considered, as some observational studies have provided evidence of their efficacy in reducing the risk of cognitive decline in comparison to no anticoagulation or warfarin therapy. Interestingly, randomized control trials do not show this same trend [73]. Catheter ablation for AF also shows some evidence towards reducing the risk of dementia, but reviews note that causation cannot be established due to the short follow-up and selection effects [72]. Some studies also delineate treatments such as Clitoria ternatea root extract for potentially treating chronic cerebral hypoperfusion and the associated cognitive decline [71]. Interventions such as wearable digital health technology (such as Apple Watches screening for rhythm abnormalities), routine cognitive testing, and reducing vascular risk factors may all also promote neurological health in the context of AF [72]. Interestingly, some studies propose the use of graph theory, multimodal brain MRI, and Artificial Intelligence to monitor and study cognitive decline in patients with cardiovascular risk factors and disease as well [70]. Ultimately, mitigating systemic vascular risk factors and optimizing cardiac hemodynamics remain foundational to preserving long-term neurological health within the heart–brain axis.

#### 3.2.3. Heart Failure and Neuroinflammation

Heart failure (HF) and neuroinflammation share a complex, bidirectional relationship capable of driving severe systemic and neurological decline. According to Toledo et al., 35% to 80% of HF patients experience some degree of cognitive impairment, spanning deficits in memory, attention, executive control, and processing speed [74]. Proposed mechanisms include chronic cerebral hypoperfusion secondary to HF and heightened sympathetic activity via increased norepinephrine release from the locus coeruleus, which induces oxidative stress in the hippocampus [74,75]. This oxidative stress alters peroxisome proliferator-activated receptor-gamma coactivator 1-alpha (PGC1-α) signaling and elevates mitochondrial DNA [75].

Neuroinflammation following HF has been extensively studied within the context of Alzheimer’s disease (AD) pathology, where amyloid-β accumulates in the brain. In rat models, HF correlates with elevated levels of beta-site APP cleaving enzyme 1 (BACE1) and soluble amyloid precursor protein (sAPP), alongside decreased levels of sAPP-α and α-secretase, shifting amyloid precursor protein processing toward amyloidogenesis and upregulating amyloid-β production [75]. The accumulated amyloid-β promotes a pathological feedback loop by suppressing Wnt signaling—which impairs long-term potentiation, neurogenesis, synaptic plasticity, and synaptic assembly in the brain, while promoting cardiac hypertrophy, adverse remodeling, and arrhythmias [74]. Furthermore, local neuroinflammation induced by amyloid-β compromises the BBB integrity, allowing brain-derived amyloid-β to enter systemic circulation and expose peripheral organs, including the heart, to toxic aggregates that further exacerbate HF and ischemic heart disease [75].

Another prominent mechanism linking HF to neuroinflammation involves the cyclic GMP–AMP synthase (cGAS) pathway, where cGAS accumulation within the subfornical organ (SFO) intensifies neuroinflammation and exaggerates the peripheral sympathetic drive [76]. In HF rat models, the presence of sodium-glucose cotransporter 2 (SGLT2) is associated with pronounced mitochondrial dysfunction and oxidative stress, whereas SGLT2 knockdown promotes cGAS ubiquitination and degradation [76]. This reduction in available cGAS dampens the Stimulator of Interferon Genes (STING) pathway, downstream nuclear factor kappa B (NF-κB) and interferon regulatory factor 3 (IRF-3) activation, and the overall inflammatory cascade [76]. Consequently, the administration of the SGLT2 inhibitor Empagliflozin has been shown to attenuate neuroinflammation and dampen sympathetic hyperactivity (measured by HRV), while providing peripheral benefits such as reduced myocardial remodeling, lessened systolic dysfunction, and decreased N-terminal pro-brain natriuretic peptide (NT-proBNP) levels [76].

The characterization of this axis by Zhang et al. also shows that the circulating mitochondria in HF rats exhibit elevated cGAS levels, impaired respiration, and high oxidative stress, which can be normalized by the cGAS inhibitor RU.521 [73]. RNA sequencing indicates that cGAS upregulates the expression of phospholipase A2 group IIA (PLA2G2A), a pro-inflammatory enzyme that facilitates arachidonic acid production and increases the Integrin-$\alpha_{v}\beta_{3}$ expression on astrocytes and microglia within the SFO, driving sympathetic hyperactivity [73]. The knockdown of PLA2G2A successfully restored the sympathetic balance, reducing renal sympathetic nerve activity (RSNA), preventing presynaptic neuronal sensitization, and ameliorating systolic dysfunction and myocardial remodeling [73].

Beyond the cGAS pathway, neuroinflammatory pathologies are mediated by distinct cellular and receptor-level shifts in the brain. Following HF induction in rats, the microglial expression of Angiotensin II Receptor Type 1a ($AT_{1a}$) increased within the hippocampus; antagonizing these receptors with Losartan reduced neuroinflammation and reduced cognitive deficits [77]. Notably, Losartan did not alter the baseline cerebral hypoperfusion or hypoxia, indicating its therapeutic effect is primarily mediated through direct anti-inflammatory pathways rather than hemodynamic normalization [77].

Insights from traditional medicine have introduced alternative multi-target therapies to interrupt this heart–brain axis. The Xijiaqi Formula, studied in HF rats, yields positive cardiac and cognitive effects by downregulating PDE4, thereby upregulating cAMP, Protein Kinase A, and cAMP Response Element Binding Protein, while increasing brain-derived neurotrophic factor (BDNF), PSD95, and Synaptin I [78]. These changes enhance synaptic regulation, dendritic growth, and general inflammation prevention, specifically inhibiting microglial and astrocytic activation while decreasing broader inflammatory mediators like TNF-α and IL-1 [78]. Molecular docking and bio-layer interferometry assays identify quercetin, kaempferol, isorhamnetin, and darutoside as the primary active compounds driving this protective mechanism of action [78].

### 3.3. Autonomic Imbalance and Dysregulation

#### 3.3.1. Sympathetic–Parasympathetic Imbalance

Autonomic imbalance is best framed as a pathophysiological state—not a single diagnosis—in which chronic SNS activation and/or parasympathetic (vagal) withdrawal reduces the flexibility of cardiovascular control and is associated with adverse outcomes across multiple diseases [79,80]. In this framing, heart rate control emerges from the dynamic interaction between acceleratory sympathetic influences and deceleratory parasympathetic influences, and the resulting changes in beat-to-beat intervals are observable as HRV [79]. Clinically, HRV indices derived from 24-h Holter ECG recordings are widely used as a non-invasive readout of cardiac autonomic modulation [81,82].

The low vagal influence can disinhibit sympathoexcitatory influences, amplifying the functional impact of the sympathetic drive and shifting autonomic regulation toward sympathetic dominance [79,83]. This imbalance has multi-system consequences spanning arrhythmias and sudden cardiac death, hypertension and vascular disease, metabolic syndrome/diabetes with cardiovascular autonomic neuropathy, neuroimmune dysregulation, and psychiatric symptom clusters characterized by an elevated heart rate and reduced HRV [81,82,84,85]. Chronic SNS activation (e.g., during prolonged stress) is explicitly described in the literature as contributing to cardiovascular issues including hypertension and HF [81]. Sympathetic hyperactivity is also coupled to inflammatory biology in ways that are relevant to chronic cardiovascular disease trajectories. Mechanistically, sympathetic neurotransmission is described as pro-inflammatory around the time of immune activation, with anti-inflammatory properties occurring in the post-activation or chronic phase of inflammation [86]. Vagal nerve activity restrains the inflammatory system and provides a concrete demonstration that subdiaphragmatic vagotomy is followed by a marked increase in NF-κB activation in the colon (measured by photon emission tomography), supporting the concept that the resting vagal tone exerts a suppressive influence over inflammatory signaling pathways [87]. Consistent with a systems-level interpretation, the low vagal cardiovascular influence is described as disinhibiting sympathoexcitatory influences, reinforcing the idea that “vagal withdrawal” can be both a loss of direct sinoatrial inhibition and a release of sympathetic dominance [83].

#### 3.3.2. Impaired Baroreflex and Heart Rate Variability

Baroreflex physiology operates as a foundational homeostatic loop: baroreceptors in the carotid sinus and aortic arch detect vessel stretch and initiate autonomic reflexes that restore blood pressure equilibrium [82]. Hypertension and diabetes frequently impair this mechanism, yielding blunted reflexes and an increased cardiovascular risk [82]. Baroreflex-related cardiovascular control is also linked to vascular tone and compliance: capacitance (through conductance inversely proportional to compliance) can induce changes in cardiac baroreflex sensitivity, and sympathetic activation acts accordingly by changing the vascular tone [81].

#### 3.3.3. Clinical Consequences

Clinically recognizable vagal impairment phenotypes occur in cardiac autonomic neuropathy (CAN), where the resting tachycardia (including >100 bpm) and/or a fixed heart rate, as well as orthostatic hypotension and exercise intolerance, are noted in the advanced stages [88]. Autonomic failure, particularly through parasympathetic dysfunction, can result in a fixed heart rate that is unresponsive to moderate exercise, stress, or sleep, indicating an almost complete cardiac denervation, a deleterious complication that can have significant phenotypical influences that would result in a reduced quality of life [89].

In parallel, parasympathetic integrity is crucial for cardiovascular recovery and health, with parasympathetic dysfunction linked to an increased risk of arrhythmias, hypertension, and HF [82]. Baroreflex dysfunction, referred to as reduced baroreflex sensitivity (BRS), is a mechanism that propagates autonomic instability. In HF, baroreflex sensitivity is reduced due to central remodeling and afferent signaling alterations, and impaired baroreflex gain results in greater arterial pressure fluctuations which has been associated with contributing to episodic hypotension, a diminished vagal tone, decreased HRV, and persistent sympathetic overdrive [80]. Stroke provides an additional neurocardiovascular phenotype in which baroreflex dysfunction is clinically meaningful: stroke patients exhibit an altered resting heart rate and blood pressure dynamics, increased variability, reduced baroreflex sensitivity, and disrupted autonomic reflexes, with impaired baroreflex function reducing cardiovascular stability and contributing to instability and stress [90]. After the acute phase of ischemic stroke, a decreased HRV and impaired cardiac baroreceptor sensitivity are linked to poor clinical outcomes, and sympathetic hyperactivity together with a decreased BRS is associated with post-stroke infections and secondary brain injury [91].

### 3.4. Inflammatory and Immune Crosstalk

#### 3.4.1. Blood–Brain Barrier Disruption

The BBB is a regulated interface whose breakdown permits circulating inflammatory signals and immune cells to access the central nervous system (CNS), providing a mechanistic route by which cardiovascular and systemic inflammation can amplify neuroinflammation [92,93]. Experimental BBB models show that pro-inflammatory cytokines can increase endothelial permeability without necessarily compromising endothelial viability, demonstrating that endothelial activation rather than endothelial death can be sufficient to compromise the barrier [93].

**Figure 4:**
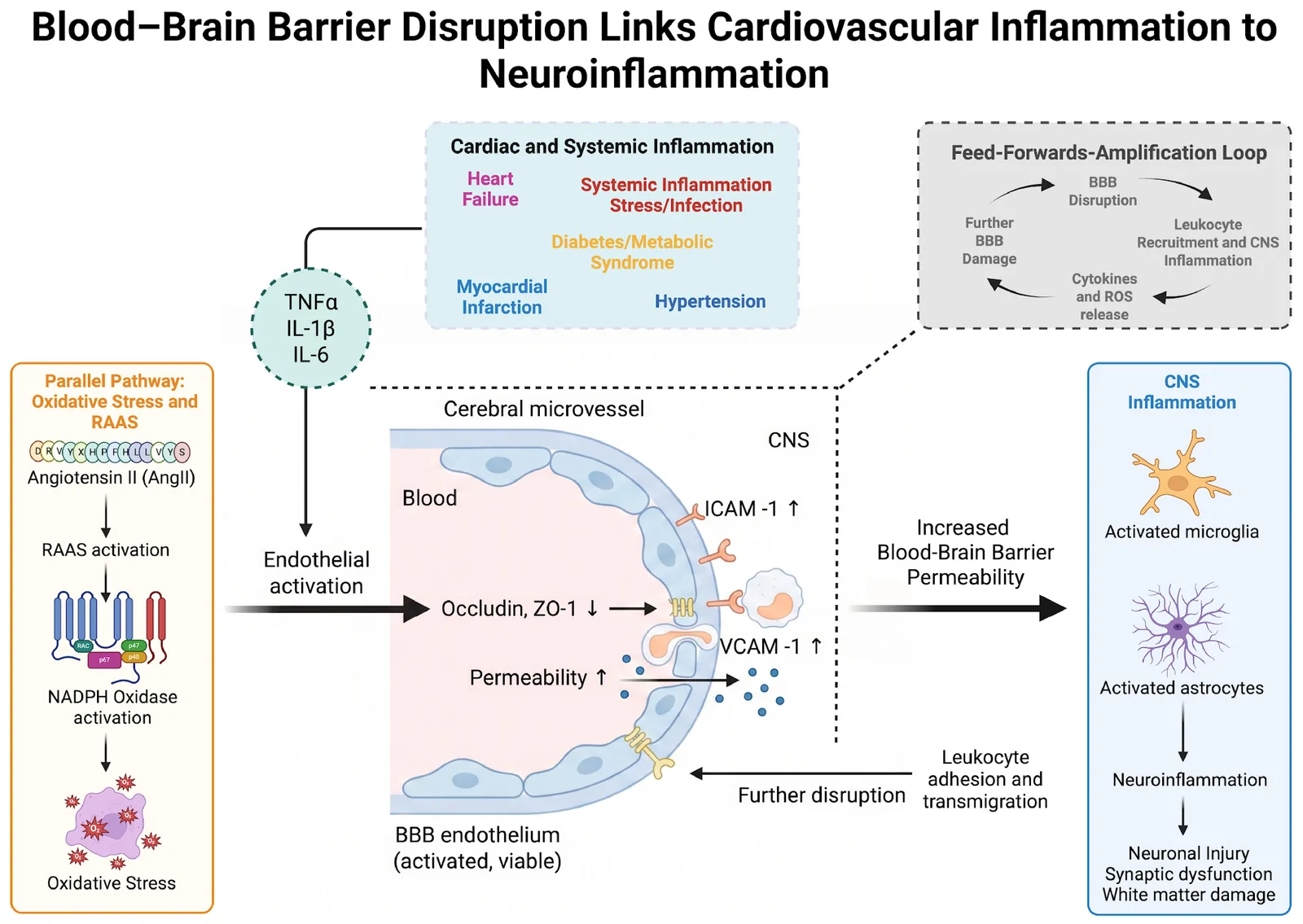
Blood–brain barrier disruption links cardiovascular inflammation to neuroinflammation. Cardiovascular and systemic inflammatory conditions promote circulating cytokines such as TNF-α, IL-1β, and IL-6, which activate cerebral microvascular endothelial cells. This increases ICAM-1 and VCAM-1 expression, reduces tight-junction proteins such as occludin and ZO-1, and enhances BBB permeability. The resulting leukocyte adhesion, transmigration, and inflammatory mediator entry into the CNS activate microglia and astrocytes, driving neuroinflammation, neuronal injury, synaptic dysfunction, and white matter damage. In parallel, activation of the RAAS and Ang II-mediated signaling stimulates NADPH oxidase activity and oxidative stress, further exacerbating endothelial dysfunction and BBB leakage. BBB disruption and neuroinflammation form a self-amplifying feed-forward loop in which cytokine release, ROS generation, leukocyte recruitment, and endothelial injury perpetuate further barrier damage and CNS inflammation. Ang II, angiotensin II; BBB, blood–brain barrier; CNS, central nervous system; ICAM-1, intercellular adhesion molecule-1; IL, interleukin; NADPH, nicotinamide adenine dinucleotide phosphate; RAAS, renin–angiotensin–aldosterone system; ROS, reactive oxygen species; TNF-α, tumor necrosis factor-α; VCAM-1, vascular cell adhesion molecule-1; ZO-1, zonula occludens-1. Created in BioRender. Singh, A. (2026) https://BioRender.com/yg2d0ra.

A recurrent theme across the literature is that inflammatory and immune cross-talk and BBB disruption are mutual: cytokines from a cardiac insult can directly increase barrier permeability, inflammatory activation increases endothelial–leukocyte interactions, and leukocyte adhesion/transmigration further disrupts the barrier [93,94]. In parallel, oxidative stress and RAAS/angiotensin II (Ang II) signaling in the cerebral circulation can produce vascular inflammation accompanied by BBB leakage, and oxidative stress scavenging attenuates both leukocyte–endothelial interactions and BBB permeability readouts (Figure 4) [95,96].

The BBB’s key functional unit is the cerebral microvascular endothelium, whose barrier properties depend on inter-endothelial junctional complexes (e.g., occludin and ZO-1) that restrict paracellular movement [97]. Consistent with this, cytokines that reduce the expression of junctional proteins are associated with an increased macromolecular diffusion across BBB endothelial monolayers [97]. Even when the BBB endothelium remains viable, it can transition to an activated inflammatory phenotype, which is particularly important for heart–brain immune cross-talk because such an activation can increase the expression of immune-recruitment pathways while simultaneously weakening barrier function [93,94]. In inflammatory CNS lesions, BBB endothelium is described in the literature as expressing cell adhesion molecules (CAMs) such as ICAM-1 and VCAM, which supports leukocyte recruitment across the vascular wall [94].

#### 3.4.2. Cytokine Signaling

TNF-α can directly increase both the transcellular and paracellular permeability across BBB endothelial monolayers [93]. Versele et al. demonstrated that TNF-α significantly increased the transcellular and paracellular passage of lucifer yellow across a BBB endothelial monolayer by 40.1% and 175.2%, respectively, compared with control in a human BBB dysfunction model [93]. More broadly, Versele et al. provided evidence that proinflammatory cytokines can induce inflammatory responses in BBB endothelial cells without altering the viability [93]. IL-1β is another pro-inflammatory cytokine that can increase the BBB permeability via a glial–endothelial coupling mechanism involving astrocyte-derived Sonic Hedgehog (SHH) signaling [98]. In a study by Wang et al., IL-1β suppressed the SHH expression in astrocytes and increased the BBB permeability by downregulating tight-junction (TJ) proteins in endothelial cells [98]. Additionally, it was demonstrated that IL-1β administration alone significantly increased the BBB permeability, and conditioned media from IL-1β-stimulated astrocytes lost the ability to increase the BBB integrity [98]. Reactivating SHH signaling (via astrocyte conditioned media, SHH itself, or purmorphamine) upregulated TJ-associated proteins, whereas the smoothened antagonist cyclopamine ablated the astrocytic effect, supporting a pathway-specific lever for BBB restoration in cytokine-driven dysfunction [98]. T-cell-linked cytokines can also weaken the BBB junctional integrity [97]. Kebir et al. found that the addition of IL-17 or IL-22 to BBB endothelial monolayers induced a marked and sustained increase in the diffusion of fluorescence-labeled albumin (BSA) [97]. This effect was dose-dependent and, for IL-17, coincided with the decreased expression of occludin and ZO-1 [97]. Rochfort et al. added to the line of evidence by demonstrating that pro-inflammatory cytokines can induce oxidative stress in brain microvascular endothelial cells in a time- and dose-dependent manner in human brain microvascular endothelial cells [99]. The same study found that TNF-α and IL-6 exposure is associated with an increased expression of the NADPH oxidase subunits gp91 and p47, with dose-dependent increases up to 2.1-fold (gp91) and up to 3.5-fold (p47) at 100 ng/mL cytokine conditions [99]. Cytokine exposure also increased the co-association of gp91 and p47 (up to 3.5-fold with TNF-α and up to 3.8-fold with IL-6), consistent with the increased assembly of the NADPH oxidase complex under inflammatory stimulation [99].

Functionally, antioxidant and NADPH oxidase-targeted interventions can attenuate cytokine-induced permeability changes and partially restore junctional protein expression [99]. ROS-depleting agents maximally attenuated TNF-α- and IL-6-mediated permeabilization by 50% and 45%, respectively [99]. Similarly, the siRNA knockdown of gp91 or p47, or the blockade of Rac1 activation, attenuated the permeabilizing effects by 47% and 53%, respectively. The targeted blockade of the same pathway partially recovered the cytokine-mediated downregulation of junctional proteins by approximately 40% for TNF-α and IL-6 [99]. Oxidative stress can also be generated at the leukocyte–endothelial interface and contribute to immune trafficking across the BBB [94]. Specifically, ROS produced by monocytes upon firm adhesion to endothelial cells can enhance monocyte migration and adhesion, providing a feed-forward mechanism whereby inflammation and immune recruitment promote further inflammation [94].

#### 3.4.3. Microglial and Cardiac Immune Activation

Cardiac immune activation from cardiac injury and HF are accompanied by (i) the remodeling of the microglial spatial and inflammatory states in discrete brain regions and (ii) the recruitment and activation of cardiac myeloid populations that shape cytokine production, chemokine release, and downstream adaptive immune responses [77,100,101]. Evidence within the literature demonstrate two empirically supported pathways: a hippocampal vascular niche enriched for vessel-associated microglia (VAM) in HF that is sensitive to AngII and AT1aR signaling [77], and a cardiac innate immune program in which CCR2-associated monocyte/macrophage recruitment and MyD88-dependent inflammatory cascades drive the cytokine and chemokine expression after cardiomyocyte injury and ischemia-reperfusion [100,102]. These pathways are linked to clinically relevant neurobehavioral domains via microglial/astrocyte remodeling and increased cytokine levels in the central amygdala (CeA), with severity-dependent correlations to the ejection fraction [101].

In HF rat models, microglial remodeling has been demonstrated as not being confined to a single autonomic node but includes hippocampal and forebrain microglial changes that co-occur with the increased cytokine readouts in the CeA [77,101]. Althammer et al. demonstrated that, in the hippocampus, HF was associated with a redistribution of microglia toward the vasculature: the proportion of VAM increased 2.1-fold in HF compared with sham, accompanied by a reduction in parenchymal microglia [77]. In parallel, HF is associated with an overall ~40% increase in VAM abundance with a simultaneous decrease in parenchymal microglia, reinforcing that HF alters the balance between vascular-associated and parenchymal microglial compartments rather than merely changing one subtype in isolation [77]. In the CeA, a region implicated in emotion and cognition, Althammer et al. demonstrated that HF induced microglial/astrocyte cell remodeling with increased cytokine secretion, linking cardiac decompensation to neuroinflammatory remodeling in the forebrain circuit that is often discussed in the context of mood and cognitive phenotypes [101]. Althammer et al. also demonstrated a temporal dynamic to this relationship: neuroinflammation-related changes in the CeA occurred with a delayed time course relative to those in the PVN and correlated with the HF severity, a pattern interpreted as potentially contributing to CI and mood disorders observed at the later disease stages in HF patients [101]. The CeA microglial remodeling was also severity-linked, with strong correlations reported between the microglial cell volume, filament length, and branch number with the percent ejection fraction [101], indicating that HF severity can influence the microglial-immune response with the brain.

### 3.5. Metabolic and Oxidative Stress

#### 3.5.1. Reactive Oxygen Species

Reactive oxygen species (ROS) are a common mechanistic link between cardiac and cerebral pathology. Multiple enzymatic sources of ROS exist, with mitochondrial electron transport chain complexes and NADPH oxidase (NOX) isoforms cited most frequently within the literature [103,104,105,106]. In the brain, NADPH oxidase and mitochondrial complexes I and III are key generators of superoxide and hydrogen peroxide [104,105], while, in the heart, xanthine oxidase, monoamine oxidase, and cytochrome P-450 are additionally implicated [104]. A critical distinction is that ROS serve a dual role in heart–brain interactions: as essential signaling molecules and as agents of oxidative damage. In the signaling capacity, ROS activate mitogen-activated protein kinases (MAPKs) and transcription factors such as NF-κB, HIF-1, and AP-1, which modulate the ion channel function, neuronal excitability, and sympathetic outflow in cardiovascular control regions of the brain [103,104]. This redox-mediated signaling is particularly relevant in the context of Ang II, which relies on ROS as second messengers to drive sympathoexcitation and blood pressure elevation [103,107,108]. In preconditioning paradigms, mitochondrial ROS also activate cell survival programs through potassium-channel-dependent mechanisms (mitoKATP and mBKCa), protecting tissues from subsequent ischemic insults [109,110].

Conversely, when ROS production is excessive or sustained—as in ischemia/reperfusion—these species damage the cellular components, trigger apoptosis, degrade the extracellular matrix, and exacerbate the infarct size in both the heart and brain [104,109]. There also exists a temporal relationship with the ROS production profiles: in ischemic reperfusion injury, ROS peak within 2–10 min of reperfusion and trigger neutrophil chemotaxis, establishing a feed-forward inflammatory loop [104]. In post-MI HF, elevated superoxide in the paraventricular and supraoptic nuclei was sustained at 2 and 4 weeks, contributing to chronic sympathetic hyperactivity [107]. In the hypertensive (mRen2)27 rat, elevated mitochondrial ROS in the dorsal medulla impaired baroreflex sensitivity chronically, an effect that was reversed by the four-week intracerebroventricular infusion of the mitochondria-targeted scavenger Mito-TEMPO [111].

#### 3.5.2. Endothelial Dysfunction

Endothelial dysfunction, particularly the BBB compromise, acts as a convergence point linking cardiac injury with cerebral pathology. The BBB breakdown has been consistently documented in the literature following cardiac ischemic reperfusion injury [112,113,114], MI [115], and viral myocarditis [116], and in the context of systemic aging [105]. In the aging brain, oxidative stress disrupts endothelial mitochondrial function, compromises the tight-junction architecture, and accelerates angiogenic failure, producing capillary rarefaction and a reduced nitric oxide bioavailability [105]. These pathological features are shared between peripheral artery disease and cerebral microvascular dysfunction, reinforcing the concept of a unified systemic microvascular aging phenotype [105]. In viral myocarditis, proinflammatory cytokines released by infected cardiomyocytes (IL-1β, IL-6, and TNF-α) activate endothelial cells and disrupt the BBB, allowing immune cell infiltration and microglial activation through NF-κB-dependent pathways, which, in turn, contributes to sympathetic overdrive and arrhythmogenesis in a self-perpetuating cycle [116].

#### 3.5.3. Mitochondrial Injury

Mitochondrial dysfunction has been demonstrated to occur in both cardiac and brain tissues and across diverse disease models, positioning mitochondria as amplifiers—and, in some cases, initiators—of the heart–brain pathology. In the heart-to-brain direction, cardiac ischemia/reperfusion caused a brain mitochondrial dynamic imbalance in rats, with a decreased expression of fusion proteins Mfn1, Mfn2, and OPA1, alongside a paradoxically increased OXPHOS complex I and III expression [112]. In this same study by Surinkaew et al., cardiac ischemic reperfusion injury also produced a BBB breakdown, brain oxidative stress, increased amyloid-β production, and brain apoptosis [112]. In an MI model, brain mitochondrial dysfunction was accompanied by dendritic spine loss, BBB breakdown, and a metabolic shift from oxidative phosphorylation toward glycolysis [113]. Jinawong et al. showed that mitochondrial fission inhibitor (Mdivi-1), fusion promoter (M1), and enalapril all improved cognitive function in MI rats, though only Mdivi-1 restored mitochondrial fusion [113].

Genetic models of cardiac mitochondrial dysfunction provide direct evidence of the heart-to-brain transmission of mitochondrial injury. In Lonp1cKO mice with HF, CBF was reduced by approximately 43% (587 ± 34 vs. 1034 ± 31 perfusion units, *p* < 0.0001), and mitochondrial complex I through V activities were significantly reduced in both the hippocampus and cortex [117]. These mice showed the upregulation of UPRmt stress markers (ATF4 and ATF5) and increased cortical oxidative stress (38.06 ± 1.8 vs. 30.09 ± 0.8) [117]. A gene expression analysis in the same model revealed the upregulation of hypoxia (Hif1a), cellular stress (Atf6), and mitochondrial biogenesis markers (Tfam, mt-COI, and mt-COII), alongside the downregulation of neurotrophic factors (Bdnf, Ngf, and Gfap) [118]. In the cardiopulmonary bypass model, Volk et al. had piglets exposed to 4 h of cardiopulmonary bypass and found that they exhibited significantly increased cerebral mitochondrial ROS and a decreased maximal oxidative phosphorylation capacity, even in the absence of local ischemia markers [119]. This finding suggests that cardiac procedures can produce direct cerebral mitochondrial injury independent of direct ischemia. In the brain-to-heart direction, Ang II-driven mitochondrial ROS production in brainstem cardiovascular nuclei have been shown to contribute to sympathetic overactivation and an impaired baroreflex in rats [106,111]. Furthermore, Lindley et al. found that the intracerebroventricular delivery of superoxide dismutase after MI reduced the neuronal activation markers and sympathetic drive in mice [107].

### 3.6. Clinical Implications

#### 3.6.1. Heart Failure and Cognitive Impairment

CI is common in HF populations and is increasingly recognized as a clinically important comorbidity with direct implications for outcomes and care delivery [120,121]. In a systematic review and meta-analysis by Cannon et al. comprising 26 studies (*n* = 4176), the estimated prevalence of CI in HF cohorts was 43% (95% CI 30–55) [120]. The reported prevalence varies widely by setting and HF severity, including reports of the highest prevalence (up to 80%) among patients hospitalized with acute decompensation [122]. CI in HF is not merely descriptive; multiple studies describe CI as an independent risk factor for death and readmission, and acute HF cohorts show higher composite events, mortality, and readmission among patients with CI compared with those without CI [121,123].

Estimates differ in incident HF, where the CI prevalence may resemble that of matched non-HF controls at the point of incident diagnosis; in the REasons for Geographic And Racial Differences in Stroke (REGARDS) study, the CI prevalence in incident HF (14.9%) was similar to the matched non-HF controls (13.4%) [124]. This incident-cohort pattern most likely suggests that much of the cognitive decline may occur after HF diagnosis rather than preceding it, which has important implications for longitudinal monitoring and prevention strategies after HF onset. Multiple reviews similarly describe CI prevalence ranges of 25–75% across population-based studies (reflecting definitional heterogeneity), with CI present in around 40% and up to 60% of elderly HF patients in some reports [121,122]. In a large multicenter Italian survey, CI was detected in about 35% of HF patients versus 29% in non-HF patients, suggesting a sizable clinical burden even when comparing across broad health-system populations [121]. Critically, CI in HF is most likely under-reported as cognitive decline is typical in normal aging. Estimates show that nearly half of CI in HF may be underdiagnosed, which can mask the risk in standard HF workflows [121].

The importance of a sufficient follow-up in HF patients is critical for CI monitoring and is highlighted by multiple studies in the literature. Among studies that followed participants for ≥1 year, 3 of 4 studies reported cognitive decline in HF, whereas only 1 of 11 studies with a follow-up <1 year reported cognitive decline [125]. Consistent with this, one review states that, over longer periods (>12 months), cognitive function tends to decline, aligning CI in HF with a progressive risk profile rather than a purely static comorbidity [125]. Large-cohort data also quantify incident-HF-associated decline. In the Atherosclerosis Risk in Communities (ARIC) study, participants who developed incident HF between visits 4 and 5 experienced greater cognitive decline between those visits compared with those who did not develop HF [126]. While HF is described as a risk factor for degenerative disorders such as AD and vascular dementia, two studies cited in one review found relative stability over short intervals in mild HF, reinforcing that clinical surveillance should be oriented to longer horizons rather than short-term stability alone [127]. Crucially, cognitive deterioration in acute HF carries a near-term prognostic signal. Unrecognized cognitive decline was associated with higher six-month mortality and hospital readmissions in one report by Dodson et al. [128]. A review by Čelutkienė et al. summarized that cognitive deterioration in acute HF has been associated with a twofold increase in 30-day death/readmissions and almost fivefold 1-year mortality rates, with such associations observed even in mild CI that frequently remains undiagnosed [122].

#### 3.6.2. Stroke and Cardiac Dysfunction

Stroke-to-cardiac coupling is a notable clinical expression of the heart–brain axis because acute brain injury can precipitate myocardial injury, arrhythmias, and transient cardiomyopathy that worsen short- and long-term outcomes [129,130,131,132,133]. Acute stroke can disrupt autonomic control, producing central autonomic dysfunction in an estimated 25–76% of patients and creating a substrate for arrhythmia, biomarker-positive myocardial injury, and cardiac complications within the first 30 days of stroke (referred to as the “Stroke–Heart syndrome” concept) [131].

AF is a commonly noted outcome in patients who experience stroke. Clinically, some important questions emerge on the stroke-to-cardiac coupling relationship: whether AF is present after an embolic-appearing or cryptogenic, how to interpret and respond to post-stroke troponin and natriuretic peptide elevations, and how lesion topography (especially insular involvement) alters the arrhythmia risk, and what intensity/duration of rhythm monitoring is likely to change the management by increasing AF detection [130,132,134,135,136]. Stroke provides an additional neurocardiovascular phenotype in which baroreflex dysfunction is clinically meaningful: stroke patients exhibit altered resting heart rate and blood pressure dynamics, increased variability, reduced baroreflex sensitivity, and disrupted autonomic reflexes, with an impaired baroreflex function reducing cardiovascular stability and contributing to instability and stress [90]. After the acute phase of ischemic stroke, thedecreased HRV and impaired cardiac baroreceptor sensitivity are linked to poor clinical outcomes, and sympathetic hyperactivity together with decreased BRS is associated with post-stroke infections and secondary brain injury [91].

Stroke-related cardiac injury is also linked to autonomic dysregulation, including a reduced parasympathetic tone and sympathetic activation with the catecholamine release via adrenal and sympathetic pathways [131]. Insular cortex lesions are singled out as a high-risk substrate: insular lesions are frequently associated with arrhythmia (e.g., AF) and abnormal arterial pressure patterns together with high catecholamine levels and the simultaneous elevations of troponin T and BNP [135]. The literature also demonstrates that stroke lateralization results in different cardiac outcomes. Right insular damage is more likely to cause cardiac lesions, especially arrhythmias, and the insular influence on the sympathetic output requires integration through hypothalamic nuclei (rather than the direct projection to sympathetic preganglionic neurons) [132]. Human intraoperative stimulation data are consistent with lateralized autonomic effects: right insular stimulation promotes sympathetic responses whereas left-sided stimulation leads to bradycardia and depressor responses [63]. In a study by Colivicchi et al., right insular damage was associated with a reduced HRV, and right insular damage with arrhythmia due to sympathetic activation is described as an adverse factor affecting 1-year prognosis [137]. A study by Ay et al. further reported that about 88% of patients with right insular damage developed a myocardial injury in the weeks after ischemic stroke, underscoring the potential scale of neurogenic myocardial involvement in that subgroup [138]. Electrocardiographic abnormalities are also common after stroke across subtypes. For instance, one study by Lavy et al. (1974) reported 67% of acute ischemic stroke patients developed ischemic/arrhythmic ECG abnormalities in the first 24 h following the insult [139]. Consistent with this, a review describes a high prevalence of ECG abnormalities in acute ischemic stroke (with the reported incidence exceeding 90%), including QTc prolongation and AF among common manifestations [132].

#### 3.6.3. Arrhythmias and Sudden Cardiac Death

A key regulator of arrhythmogenesis in the heart–brain connection is autonomic imbalance, with sympathetic activation increasing automaticity, shortening the refractory period, and promoting afterdepolarizations, thereby increasing the susceptibility to atrial and ventricular arrhythmias [82]. Cardiac arrhythmias occurring in the context of heart–brain interactions span a wide spectrum, from ventricular tachycardia and ventricular fibrillation driven by sympathetic overactivation [140,141], to bradyarrhythmias and asystole mediated by parasympathetic surges during epileptic seizures [142], to repolarization abnormalities (QT prolongation, T-wave changes, and ST-segment alterations) characteristic of acute cerebrovascular events such as subarachnoid and intracerebral hemorrhage [143]. Sudden cardiac death (SCD) represents the most severe outcome of this dysregulation, claiming approximately 310,000 lives annually in the United States alone [141], with roughly two-thirds of cases occurring in individuals without prior recognized cardiac disease [141]. The heart–brain connection operates through a bidirectional neural loop—termed the cardiac–cerebral reflex—linking cardiac sensory afferents with the Central Autonomic Network, including the nucleus tractus solitarius and insular cortex [50,144]. Across diverse neurological triggers—stroke, epilepsy, subarachnoid hemorrhage, mental stress, circadian disruption, and inherited channelopathies—the unifying pro-arrhythmic mechanism is autonomic imbalance, specifically excess sympathetic discharge coupled with vagal withdrawal, which shortens the ventricular refractoriness and promotes re-entrant arrhythmias [140,144]. The specific arrhythmia type is predictable from the autonomic signature of the underlying condition: sympathetic-dominant states produce ventricular arrhythmias [140], parasympathetic-dominant states produce bradyarrhythmias [141,142], and mixed activation generates AF [141].

#### 3.6.4. Psychiatric Comorbidities

Depression and anxiety emerge in cardiovascular disease through a bidirectional relationship driven by shared pathophysiological substrates [145,146,147]. Prevalence rates are high—approximately 19.8% for depression in coronary artery disease and 24.7% in heart failure [145], with psychiatric symptoms arising both as precursors to cardiac events and as consequences of acute cardiac illness such as myocardial infarction [145]. Vulnerability is moderated by gender (women post-MI are disproportionately affected) [145], disease severity (mood disorder prevalence rises with advancing cardiovascular disease) [147], and personality factors including Type-D personality and hostility [148]. Once established, depression tends to be chronic and self-reinforcing: cardiac deterioration worsens psychiatric symptoms, which, in turn, accelerate cardiovascular decline through both behavioral pathways (medication non-adherence, physical inactivity, and smoking) [145] and direct pathophysiology [149]. The mechanisms connecting psychiatric comorbidities to cardiovascular function converge on a core triad of autonomic nervous system dysfunction (reduced HRV, and sympathetic overdrive) [146,150], HPA axis dysregulation (elevated cortisol and catecholamines) [145], and systemic inflammation (elevated CRP, IL-6, and pro-inflammatory status) [145,146], supplemented by endothelial dysfunction and platelet hyperactivation that promote atherosclerosis and thrombosis [146,151].

### 3.7. Diagnostic and Biomarker Insights

#### 3.7.1. Neuroimaging and Cardiac Imaging

Neuroimaging and cardiac imaging provide diagnostic and biomarker insights primarily through machine-learning-driven extraction of quantitative features from imaging data, enabling classification accuracies ranging from 83.6% to 95.4% across various conditions including the cardiovascular burden [152], neurodegenerative diseases [153], and adolescent depression [154]. Prognostic biomarkers derived from imaging demonstrate a strong predictive performance when externally validated: proteomic–imaging integration across over 50,000 UK Biobank participants identified 404 cardiac and 76 brain MRI-associated proteins, over 90% of which are novel candidate biomarkers with genetically supported causal roles in disease [155]. Multimodal approaches—combining MRI with PET, integrating functional and structural neuroimaging, or interpreting one modality through another—consistently outperform single-modality analyses [156].

A notable finding across the literature is the bidirectional connection between cardiac and brain imaging biomarkers. Brain structural changes such as hippocampal volume loss and insular thinning are significantly associated with the cardiovascular genetic risk [152], and 37 circulating proteins correlate with imaging-derived phenotypes in both the heart and brain [155].These cross-organ findings, supported by shared vascular and inflammatory pathways [155], suggest that imaging in one organ system can inform the disease risk in the other. However, the strength of the evidence varies: externally validated studies with large samples yield more conservative performance estimates than smaller studies relying solely on internal cross-validation [153], and generalizability remains constrained by the predominantly European-ancestry composition of the largest cohorts [155].

#### 3.7.2. Circulating Biomarkers

Circulating cardiac biomarkers—particularly NT-proBNP and high-sensitivity troponin (hs-cTnT/hs-cTnI)—are consistently associated with structural brain damage and cognitive decline across diverse populations. Higher NT-proBNP levels are linked to reduced cortical thickness [157], lower grey matter volume [158,159], greater white matter hyperintensity burden [160,161], and silent brain infarcts [160], while elevated troponin is independently associated with neuroaxonal injury as measured by a neurofilament light chain [162], white matter lesion progression [160], and cerebrovascular disease in CI and dementia [163]. Both biomarkers predict longitudinal cognitive decline across memory, executive function, processing speed, and global cognition [164,165,166,167], with mediation analyses indicating that brain free water and structural brain changes fully mediate the pathway from cardiac biomarkers to cognitive deterioration [157,165]. Additional markers including GDF-15 [164,165], BDNF (inversely correlated with BNP in HF) [168], and galectin-3 [169] provide complementary but less extensively replicated findings, while large-scale proteomics has identified hundreds of novel-shared molecular candidates with genetic evidence supporting causality for over 63% of them [155].

Despite robust associations at the population level, the diagnostic performance of these biomarkers for the individual-level prediction of cognitive decline remains modest, with AUC values ranging from 0.58 to 0.71 [164]. Diagnostic utility is stronger in acute clinical contexts: BNP achieves an AUC of 0.86 for post-stroke mortality prediction [170], and troponin combined with NT-proBNP detects stress-induced cardiomyopathy after subarachnoid hemorrhage with 100% sensitivity [171]. Age is a key effect modifier, as cardiac biomarkers are associated with structural brain changes across the age spectrum but with measurable CI only in older individuals [158]. Troponin and natriuretic peptides appear to index partially distinct pathways—troponin reflecting myocardial injury with stronger links to vascular brain damage [162], and NT-proBNP capturing broader neuroa sdegeneration and hemodynamic compromise [157] —suggesting they are best used in combination for comprehensive heart–brain risk assessment rather than as standalone diagnostic tools.

#### 3.7.3. Heart Rate Variability and Autonomic Testing

HRV serves as a consistent, non-invasive biomarker of the heart–brain axis integrity across a broad spectrum of conditions. A reduced HRV is uniformly associated with worse outcomes, including an accelerated cognitive decline (0.06–0.07 SD per decade faster in individuals with low vagal HRV [172]), an increased stroke risk (HR 1.4–1.7 for time-domain parameters [173]), greater dysautonomia severity in Parkinson’s disease [174], and an elevated psychiatric symptom burden [175]. Prognostically, HRV achieves a high specificity (up to 99%) for post-myocardial infarction risk stratification [176], predicts dementia incidence with HR of 2.2–3.2 for lowest-quartile standard deviation of NN intervals (SDNN) [176], and detects persistent autonomic dysfunction after traumatic brain injury beyond subjective symptom resolution [177,178]. Multimodal approaches combining HRV with neuroimaging—such as HRV-BOLD synchronization in Parkinson’s disease [175] and EEG-HRV coupling in Alzheimer’s disease [179]—show enhanced discriminative power over HRV alone.

However, HRV’s clinical utility as a standalone diagnostic tool is constrained by several factors. The effect sizes for the cognitive prediction are modest (cross-sectional r = 0.25) [180], the sensitivity in cardiovascular risk models is limited, and subclinical autonomic neuropathy may confound resting HRV in medically complex populations [181]. HRV is not a specific marker of sympathetic activity and is substantially influenced by age, respiration, medication, and recording conditions. The marked heterogeneity in acquisition protocols across studies remains the most significant barrier to standardized clinical implementation. The question of causality—whether autonomic dysfunction drives brain pathology or vice versa—remains unresolved, though longitudinal evidence linking midlife HRV to subsequent cognitive decline supports at least partial directionality from autonomic dysfunction to neurodegeneration.

### 3.8. Future Directions and Research Gaps

#### 3.8.1. Mechanistic Studies Linking Heart Failure and Neurodegeneration

Mechanistic studies linking HF-associated neurodegeneration emphasize reduced the CBF and the dysfunction of the neurovascular unit and BBB, while explicitly noting that the HF–AD relationship remains largely unclear and that causality is unproven in the absence of dedicated cognitive endpoints in randomized trials [182,183,184]. Translational opportunities highlighted in the literature include SGLT2 inhibitors as potential modulators of oxidative stress and neuroinflammation, with observational evidence suggesting a reduced risk of dementia but limited HF-specific cognitive trial data. Neuromodulation, particularly vagus nerve stimulation (VNS), also represents a promising therapeutic strategy; however, large randomized controlled trials have yielded largely negative results, highlighting the need for the optimization of the stimulation dose, an improved assessment of autonomic target engagement, and better-defined clinical end points [75,182,183,185]. One review explicitly aims to explain how HF contributes to the development of AD, focusing mainly on the reduced CBF and dysfunction of the neurovascular unit [184]. Within this framing, neurons in HF are described as chronically exposed to insufficient blood supply, with a lack of energy leading to acidosis and oxidative stress, and brain acidosis is associated with aggregation of altered tau and amyloid-β [184]. The BBB is positioned as a central mechanistic interface, and an important mechanism in HF may be the impaired amyloid-β clearance across the BBB and that hypoxia can cause a BBB breakdown that impairs clearance [184]. Mechanistically, amyloid-β clearance across the BBB occurs via transcytosis through BBB cells and via phagocytosis by microglia, while activated microglia may lose their ability to phagocytose amyloid-β during disease progression [184]. Inflammatory mechanisms are linked to BBB and glial phenotypes: systemic inflammation is associated with circulating proinflammatory cytokines that impair the brain microvascular endothelium, increase the BBB permeability, and shift astrocytes and microglia toward pro-inflammatory phenotypes, with the M1-shifted microglia secreting cytokines that contribute to neurodegeneration pathways including tau hyperphosphorylation and amyloid-β oligomerization [125,183].

Additional pathways connect hypoperfusion, BBB permeability, and amyloidogenesis. Hypoperfusion-induced hypoxia is described in the literature as inducing HIF-1 and increasing the VEGF expression, increasing the BBB permeability by destroying tight junctions, while ROS are described as increasing the BBB permeability via MMP activation and oxidative damage to cellular molecules [121]. Oxidative damage is also described as activating BACE-1 with an increased APP and amyloid-β synthesis being associated with the downregulation of BACE-2 contributing to the reduced degradation of amyloid-β precursor proteins [121]. The framework is described as self-reinforcing: the reduced CBF and chronic perfusion can decrease the ability of glial cells to eliminate amyloid-β, while amyloid-β can aggravate pathological alterations, forming a vicious circle described as ultimately leading to CI in HF [121].

#### 3.8.2. Longitudinal Clinical Studies on Heart–Brain Interactions

##### HF and Cognitive Decline

One of the most directly quantified prospective data in the literature hails from the Cardiovascular Health Study (CHS), a community-based prospective cohort of 4864 older adults without baseline HF and free of clinical stroke at enrollment, with annual cognition assessments from 1989/1990 through 1998/1999 [186]. In adjusted models, incident HF diagnosed at age 80 was associated with a substantially greater five-year decline in global cognition (3MSE): 10.2 points from age 80 to 85 (95% CI 8.6–11.8) after incident HF versus 5.8 points (95% CI 5.3–6.2) without HF [186]. This association was stronger at older ages than at younger ages within the evaluated range and did not vary significantly by AF status or by the reduced versus preserved ejection fraction category [186]. The CHS data also illustrate cognitive-domain specificity: the decline in Digit Symbol Substitution Test (DSST) score was not significantly different after the incident HF compared with no HF, and the investigators stated they were unable to conclude an association between incident HF and decline in psychomotor processing speed [186]. A second prospective example in the literature comes from the ARIC study, where a composite z-score across three neurocognitive tests was standardized to a reference visit and then compared over a 15-year interval [126]. Participants who developed HF over the 15-year period exhibited a greater decline versus those who did not, with an adjusted difference of −0.07 (95% CI −0.13 to −0.01) in standardized units over 15 years [126]. Longitudinal observations from the ARIC study also link the prevalent HF to a higher CI prevalence at follow-up, including the higher prevalence of dementia (adjusted RRR 1.60, 95% CI 1.13–2.25) and mild CI (RRR 1.36, 95% CI 1.12–1.64) compared with participants without HF, and an increased prevalence of CI (odds ratio 1.62, 95% CI 1.48–1.79) [126].

##### AF and Dementia Risk

Multiple longitudinal studies in the literature support an association between AF and subsequent dementia, including evidence that the association can persist after accounting for clinically recognized stroke. In the UK Whitehall II study (ages 45–69 years), AF was significantly associated with a higher risk of incident dementia (HR 1.87, 95% CI 1.37–2.55), and a longer exposure to AF was associated with faster cognitive decline; taking incident stroke into account did not significantly alter results [187]. The follow-up and event surveillance in persons who experienced AF are illustrated by Adult Changes in Thought (ACT) cohort analyses (total *n* = 3045; 20,806 person-years follow-up, including 2150 person-years with AF) [188]. In that cohort, the adjusted HR for all-cause dementia associated with AF was 1.38 (95% CI 1.10–1.73), and the risk of possible or probable AD was also higher (adjusted HR 1.50, 95% CI 1.16–1.94) [188]. During follow-up, 572 participants (18.8%) developed dementia, including 449 (14.7%) with possible or probable AD [188].

Stroke-stratified analyses from ACT illustrate a potential stroke-independent component: the HR for dementia associated with AF was 1.32 (95% CI 0.85–2.07) among those who experienced a stroke during follow-up versus 1.40 (95% CI 1.08–1.82) among those who did not [188]. The evidence within the literature also highlights effect modifications by study population. A systematic review of 15 longitudinal population-based studies (46,637 participants) reported that the AF–incident dementia association was mainly evident in studies focusing solely on patients with stroke (HR 2.4, 95% CI 1.7–3.5), while remaining marginal in broader populations (HR 1.6, 95% CI 1.0–2.7) [187]. Another meta-analysis of eight longitudinal studies without acute stroke at baseline (77,668 participants) reported AF was independently associated with an increased dementia risk (HR 1.4, 95% CI 1.2–1.7) [187]. A larger systematic review including 21 cross-sectional or longitudinal studies similarly reported AF associated with a >2-fold increased risk after stroke (HR 2.7, 95% CI 1.8–4.0) and a weaker but still significant association when restricted to participants without a history of stroke (HR 1.4, 95% CI 1.1–1.7) [187]. Several longitudinal and registry comparisons suggest potential risk modification with AF treatment strategies. A Swedish patient registered a study reported anticoagulant-treated patients had a 29% lower risk of dementia than those without anticoagulant treatment [187]. Another community-based cohort reported warfarin therapy was associated with a 20% reduction in dementia risk over five years of follow-up [187]. In the Intermountain Atrial Fibrillation Study, 0.2% of AF ablation patients developed AD after yellow years of follow-up compared with 0.9% in unoperated AF patients and 0.5% in patients without AF [187]. In summary, neuroimaging evidence provides a plausible substrate for cognitive decline beyond clinically overt stroke: high-resolution MRI studies from the Swiss-AF cohort reported silent brain lesions in as many as 40% of AF patients [176].

##### HRV and Cognition

Across longitudinal studies of autonomic function, several cohorts link baseline HRV indices to subsequent cognitive decline, but the associations are sensitive to the HRV metric used, age strata, and the cognitive outcome selected [176]. In the Pravastatin in the Elderly at Risk (PROSPER) study, HRV from a 10-s measure was assessed by SDNN, and the lower-baseline SDNN predicted a greater decline in the Letter–Digit Coding test after the correction for confounders [176]. In Whitehall II, baseline HRV indices did not predict the cognitive performance at follow-up levels, but lower SDNN, HF, and LF predicted greater decline on the Mill Hill test after adjustment for demographics, expressed as odds ratios for being in the worst quintile of change [176]. For the dementia incidence, Framingham Offspring findings show an age-modified pattern: HRV was not associated with dementia risk across the whole cohort, but a lower SDNN and root mean square of the successive differences (RMSSD) predicted a higher dementia incidence in participants aged ≥60 at baseline after the adjustment for confounders [176]. Two additional longitudinal approaches highlight the value of dynamic or nontraditional autonomic markers. A challenge-based study (*n* = 71; follow-up mean 2.8 years) reported that, among those with mild CI at baseline, a greater HRV response to a sympathetic challenge predicted a greater episodic memory decline, whereas a greater HRV response to a parasympathetic challenge predicted less executive-function decline [189]. In the Multi-Ethnic Study (MESA)-Sleep study, a greater heart rate fragmentation was associated with worse cognitive performance at follow-up and steeper cognitive decline, while traditional HRV indices displayed no such associations [176].

##### Precision Medicine Approaches

The precision-medicine agenda in neurocardiology is motivated by the observation that labeled syndromes can contain biologically distinct subgroups, illustrated by recent efforts to stratify a highly heterogeneous HFpEF cohort into more homogeneous molecular strata using unsupervised similarity-network fusion [190]. Precision approaches are also supported by evidence that cardiac biomarkers can map onto cognitive phenotypes (e.g., higher NT-proBNP and GDF-15 in a cognitive dysfunction group, with inverse correlation of GDF-15 with MMSE) [191].

##### HF Phenotyping and Patient Stratification

HFpEF identification is a multi-stage process in which no single criterion alone is sufficient, including quality-controlled LVEF thresholds and natriuretic peptide thresholds aligned to ESC recommendations, and the validated H_2_FPEF score when imaging is unavailable [191]. Thus, a multi-omics classifier was utilized as identifying asymptomatic individuals at risk with an average of 6.3 years before symptom onset with an ROC AUC 0.929, with 77.9% of those who subsequently developed HFpEF, asymptomatic at the time of recruitment, and multi-omics data acquisition [191]. Within the established HFpEF, an unsupervised SNF clustering of a cohort of *n* = 33,480 was demonstrated to yield six distinct phenotype subgroups with unique omics profiles, including a high-risk cluster with 65.2% mortality and mean BMI 34.9 kg/m^2^ [191].

##### Multi-Omics Biomarkers

A UK Biobank-based multitask deep-learning framework (CardiOmicScore) was employed to learn disease-specific proteomic and metabolomic risk scores from 2920 proteins and 168 metabolites and using Cox proportional hazards models combining PRS, MetScore/ProScore, and clinical risk factors to predict cardiovascular disease onset [192]. ProScore and MetScore were reported as strong sole predictors (C-index ranges between 0.69–0.82 and 0.64–0.74) and as enhancing risk predictors of up to 15 years before disease onset when combined with clinical data, while PRS alone was found to have limited predictive capacity (C-index range 0.52–0.60) [192].

##### Digital Phenotyping

Wearable-derived phenotyping is supported by evidence that high-resolution, context-aware heart-rate dynamics can outperform typical summary measures like resting heart rate for predicting modifiable cardiometabolic risk markers, with an average improvement in the Brier Skill Score of 52.3% [193]. Activity-state-specific dynamics in the literature are described as containing distinct information about the cardiometabolic risk type, with sedentary-state dynamics being most predictive of lipid abnormalities and active-state dynamics most predictive of blood-pressure abnormalities [193]. Another report describes high-resolution wearable features as improving the Brier score by 17.9% and 7.36% over baselines based on age/sex and resting heart rate, and as better capturing subtle dynamics related to genomic risk (11.9–22.0% improvement in Brier scores) [193].

##### Neuromodulation Personalization

**Figure 5:**
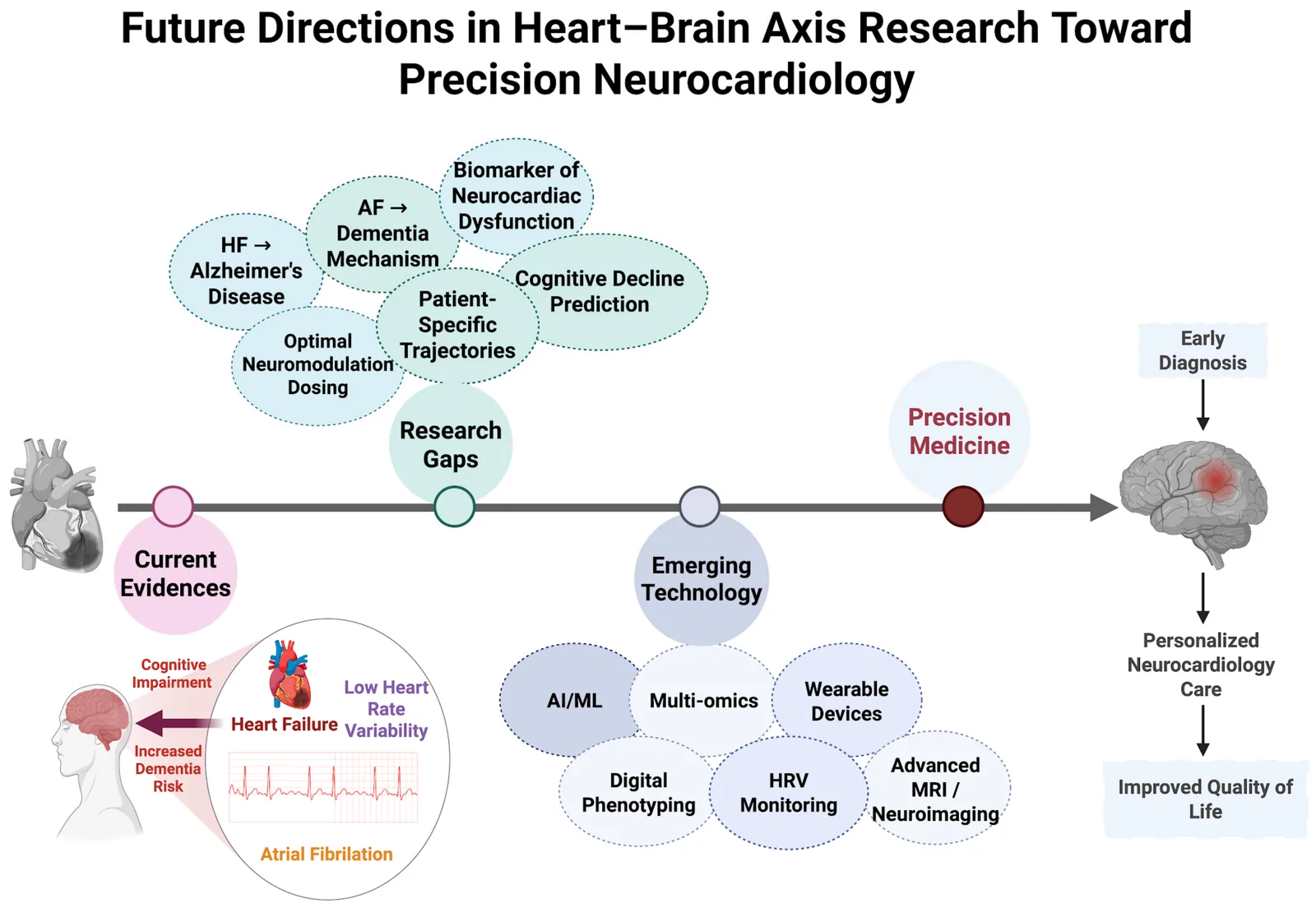
Schematic summary of current evidence linking cardiovascular dysfunction, including heart failure, atrial fibrillation, and reduced heart rate variability, with cognitive impairment and dementia risk. Key knowledge gaps include disease mechanisms, neurocardiac biomarkers, cognitive decline prediction, patient-specific trajectories, and neuromodulation dosing. Emerging technologies such as multi-omics, AI/ML, wearable devices, digital phenotyping, HRV monitoring, and advanced neuroimaging may help address these gaps and support precision medicine approaches. These advances aim to enable earlier diagnosis, personalized neurocardiology care, and improved quality of life. AD, Alzheimer’s disease; AF, atrial fibrillation; AI/ML, artificial intelligence/machine learning; HRV, heart rate variability; MRI, magnetic resonance imaging. Created in BioRender. Singh, A. (2026) https://BioRender.com/6a56bms.

Preferential efferent versus afferent fiber activation through neuromodulation personalization depends on the electrode shape/orientation and on stimulation frequencies, pulse widths, and currents, implying multidimensional dosing [194]. Clinical vagal nerve stimulation (VNS) applications use moderate intensities that cause the mild-to-undetectable slowing of heart rate and can help modulate HRV depending on the target location and stimulation parameters, but an early study found that a strong VNS (above 100% sensory thresholds) would induce AF, motivating moderate intensities (less than 80% of thresholds) [195]. Although considerable progress has been made in elucidating the mechanisms linking cardiovascular and neurological dysfunction, important questions regarding causality, risk prediction, biomarker discovery, and therapeutic optimization remain unresolved. Figure 5 provides an overview of the current evidence, major knowledge gaps, emerging technologies, and precision medicine opportunities that are expected to drive future advances in heart–brain axis research.

## 4. Conclusions

### 4.1. Summary of Key Mechanisms

#### 4.1.1. Mechanism 1: Neural and Autonomic Regulation

The heart–brain axis operates via a bidirectional network integrating central autonomic networks, peripheral pathways, and the intrinsic cardiac nervous system (“little brain”). Rest is dominated by vagal parasympathetic control, which stabilizes cardiac automaticity, while the sympathetic outflow driven by the RVLM increases the heart rate and contractility via norepinephrine and $\beta_{1}$-adrenergic receptors. Chronic autonomic imbalance—characterized by sympathetic overactivation and vagal withdrawal—impairs BRS, reduces HRV, promotes arrhythmogenesis, and accelerates HF and secondary cognitive decline.

#### 4.1.2. Mechanism 2: Hemodynamic Coupling and Cerebral Perfusion

CBF is directly coupled to the cardiac output, exerting an independent influence on the cerebral perfusion velocity beyond MAP during hypovolemia or physiological stress. While healthy individuals rely on cerebral autoregulation to shield the brain from systemic volatility, this buffering mechanism progressively declines with normal aging, heart failure, and endothelial dysfunction. Consequently, brain perfusion becomes heavily flow-coupled to cardiac efficiency, leading to a predictable decline in CBF that targets vulnerable watershed zones and the temporal lobes, accelerating structural neural degeneration.

#### 4.1.3. Mechanism 3: Heart-to-Brain Afferent Feedback and Interoception

The heart serves as an active interoceptive sensory organ, continuously transmitting mechanical pressure, stretch, and chemical signals back to the central nervous system. This feedback loop is structurally asymmetrical, as the vagus nerve consists of approximately 80% afferent sensory fibers. These ascending signals are processed by the nucleus of the solitary tract in the medulla and relayed to the subcortical and frontocortical regions, including the insular cortex, where they shape homeostatic autonomic reflexes, executive functions, and flexible emotional processing.

#### 4.1.4. Mechanism 4: Inflammatory and Immune Cross-Talk

Systemic and central immune activation provides a critical communication channel during acute and chronic pathology. Acute brain injuries and sympathetic overactivation polarize microglia and astrocytes toward pro-inflammatory states, releasing cytokines (such as TNF-α, IL-1β, and IL-6) that degrade cardiac tissue. Conversely, HF drives localized neuroinflammation in the SFO and CeA via cGAS-STING-NF-κB and PLA2G2A-integrin $\alpha_{v}\beta_{3}$ signaling, which exaggerates peripheral sympathetic drive and shifts amyloid precursor protein processing toward amyloidogenesis.

#### 4.1.5. Mechanism 5: Blood–Brain Barrier Disruption

The BBB acts as the primary interface regulating the heart–brain immune crosstalk. Systemic inflammation and circulating cytokines (TNF-α, IL-1β, IL-17, and IL-22) disrupt endothelial tight-junction complexes (such as occludin and ZO-1), increasing paracellular and transcellular barrier permeability without altering the cell viability. Endothelial activation additionally upregulates cell adhesion molecules (ICAM-1 and VCAM-1), promoting leukocyte recruitment, adhesion, and transmigration into the central nervous system, establishing a self-amplifying feed-forward loop of neuroinflammation.

#### 4.1.6. Mechanism 6: Reactive Oxygen Species and Endothelial Dysfunction

ROS and mitochondrial damage function as a final common molecular pathway driving neurocardiac degradation. Cardiac ischemia/reperfusion injury and heart failure promote a central mitochondrial dynamic imbalance, decreasing fusion proteins (Mfn1, Mfn2, OPA1) and upregulating oxidative stress markers and NADPH oxidase subunits (gp91 and p47). This accumulation of ROS drives dendritic spine loss, compromises BBB integrity, induces endothelial dysfunction via eNOS uncoupling, and sustains chronic sympathetic overactivation in cardiovascular control centers.

### 4.2. Clinical Relevance

The mechanisms of the heart–brain axis do not operate in isolation but form a self-reinforcing pathological matrix. Understanding these interactions is key to identifying the most effective therapeutic targets. Pathological states often involve the simultaneous failure of multiple feedback loops. Therefore, integrated heart–brain care requires interdisciplinary cooperation among cardiologists, stroke specialists, other specialists, and primary care physicians to ensure the optimal treatment during acute events and long-term care, alongside standardized post-stroke care concepts with the multidisciplinary collaboration and coordination of care using shared tools.

## Data Availability

No new data were created or analyzed in this study. Data sharing is not applicable to this article.
